# Systematic trait dissection in oilseed rape provides a comprehensive view, further insight, and exact roadmap for yield determination

**DOI:** 10.1186/s13068-022-02134-w

**Published:** 2022-04-19

**Authors:** Huabing Liang, Jiang Ye, Ying Wang, Xinfa Wang, Xue-Rong Zhou, Jacqueline Batley, Graham J. King, Liang Guo, Jinxing Tu, Jiaqin Shi, Hanzhong Wang

**Affiliations:** 1 Oil Crops Research Institute of the Chinses Academy of Agricultural Sciences, Key Laboratory of Biology and Genetic Improvement of Oil Crops, Ministry of Agriculture, Wuhan, China; 2 National Key Laboratory of Crop Genetic Improvement, Huazhong Agricultural University, Wuhan, China; 3 Commonwealth Scientific & Industrial Research Organisation (CSIRO) Agriculture & Food, Canberra, ACT Australia; 4 School of Biological Sciences, The University of Western Australia, Crawley, WA 6009 Australia; 5 Southern Cross Plant Science, Southern Cross University, Lismore, NSW Australia

**Keywords:** *Brassica napus*, Yield determination, Segregation distortion, Residual heterozygosity, Trait-QTL network, Positive or negative pleiotropy, Target or candidate genes, Trade-off

## Abstract

**Background:**

Yield is the most important and complex trait that is influenced by numerous relevant traits with very complicated interrelations. While there are a large number of studies on the phenotypic relationship and genetic basis of yield traits, systematic studies with further dissection focusing on yield are limited. Therefore, there is still lack of a comprehensive and in-depth understanding of the determination of yield.

**Results:**

In this study, yield was systematically dissected at the phenotypic, genetic to molecular levels in oilseed rape (*Brassica napus* L.). The analysis of correlation, network, and principal component for 21 traits in BnaZN-RIL population showed that yield was determined by a complex trait network with key contributors. The analysis of the constructed high-density single nucleotide polymorphism (SNP) linkage map revealed the concentrated distribution of distorted and heterozygous markers, likely due to selection on genes controlling the growth period and yield heterosis. A total of 134 consensus quantitative trait loci (QTL) were identified for 21 traits, of which all were incorporated into an interconnecting QTL network with dozens of hub-QTL. Four representative hub-QTL were further dissected to the target or candidate genes that governed the causal relationships between the relevant traits.

**Conclusions:**

The highly consistent results at the phenotypic, genetic, and molecular dissecting demonstrated that yield was determined by a multilayer composite network that involved numerous traits and genes showing complex up/down-stream and positive/negative regulation. This provides a systematic view, further insight, and exact roadmap for yield determination, which represents a significant advance toward the understanding and dissection of complex traits.

**Supplementary Information:**

The online version contains supplementary material available at 10.1186/s13068-022-02134-w.

## Background

Yield, which usually refers to biomass or seed yield, is the most important trait of crops, such as wheat, rice, maize, soybean, peanut, cotton, and oilseed rape [1]. For example, crop straw can be used to generate heat and electricity [2]. The grains of cereal crops (including wheat, rice, maize, etc.) are the main source of starch (the first nutrient for human), and can also be used to produce bioethanol [3]. The seeds of rapeseed, soybean, and other oil crops can be supplied for producing edible oil and biodiesel [4]. With the rapid increase in the global population, there is an urgent need to increase crop yield to meet the human demand for food and energy [5]. However, yield is also the most complex trait, as it is a composite outcome of numerous contributing traits, as well as their interactions [6]. Specifically, yield is directly determined by its multiple components with a trade-off effect between them, e.g., seed number and size [7]. This means that a change in one component for yield often causes a change in other components in an opposite direction. The trade-off among yield components is generally explained by the competition among sinks (negative feedback) due to limited resources [8]. In addition, yield is indirectly affected by numerous yield-related traits in either a positive or negative direction through undetermined mechanisms [9]. These may include growth period (e.g., flowering and maturity time), plant architecture (e.g., plant height and branch number), and resistance to biotic (e.g., disease, pest, and weed) and abiotic (e.g., drought/water logging and hot/cold) stress. Therefore, characterizing the complex relationships between yield and its components or related traits is the key to understanding what and how yield is determined. This is important not only for the evolution and physiology of plants [10, 11], but also for crop genetics and breeding [12].

**Fig. 1:**
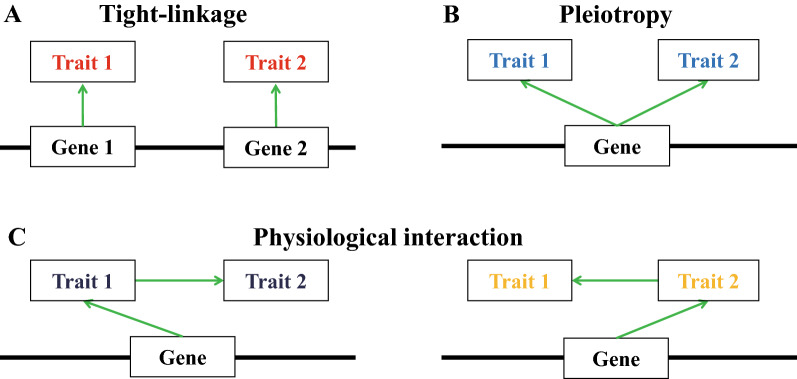
Several putative models underlying the co-localization of QTL for two traits. The first and second models (**A** and **B**) are tightly linked and generally called pleiotropy, which are easily understood. The third (**C**) is physiological interaction: one gene can indirectly affect a trait by regulating another trait

Previous studies have revealed the phenotypic correlation between yield and its components or related traits in various crops, such as rice [13], wheat [14], maize [15], soybean [16], peanut [17], and rapeseed [1]. Recently, a large number of studies have identified the underlying QTL for yield, and genome-wide QTL co-localization between yield and its components or related traits was found in crops, such as rice [18, 19], wheat [20], maize [15, 21, 22], soybean [16, 22, 23], cotton [24, 25], and rapeseed [9, 26]. However, the underlying genetic basis for phenotypic correlation and QTL clustering between yield traits is basically unclear. Theoretically, the phenotypic correlation and QTL colocalization between traits mechanistically result from either genetic linkage or pleiotropy [27]. Genetic linkage means that the genes for different traits are physically close to each other (Fig. 1A). Pleiotropy refers to the effect of a locus on two or more traits (Fig. 1B). In addition, pleiotropy may be due to physiological interactions among traits in which one trait acts at “upstream” of another (Fig. 1C). Although a few of these QTL clusters for yield traits have been further dissected to specific loci/genes [19, 28, 29], the exact relationship between the relevant traits has not been characterized.

In summary, although a large number of studies have reported the phenotypic relationship and genetic basis of yield traits, systematic research with further dissection focusing on yield is rarely performed. Therefore, there is still a lack of a comprehensive and in-depth understanding of the determination of yield. Oliseed rape is an important crop widely planted around the world for multiple purposes, including oil, fodder, and food [30]. With the successful application of catalysts in the production of biodiesel from vegetable oil [31], rapeseed also serves as a main biodiesel resource in Europe [32]. With the increasing demand for edible vegetable oil and biofuel, it is urgent to improve the seed yield of oilseed rape [33]. In the current study, taking oilseed rape as an example, yield was systematically dissected at the phenotypic, genetic, and molecular levels, with an emphasis on its complex relationship with other contributing traits. In particular, four representative hub-QTL clusters were dissected to specific loci/genes, and the causal trait relationship was revealed for the first time. The phenotypic, genetic, and molecular dissecting results were highly consistent, which provided comprehensive and further insights into yield determination.

## Results

### Analysis of phenotypic relationships revealed an integrated trait network with key factors to determine yield

In the BnaZN-RIL population and its two parents that were planted in six environments, 21 yield-related traits were investigated. The two parents Zhongshuang11 and No.73290 showed significant differences for 19 out of the 21 investigated traits in at least one environment (Additional file 8: Table S1). The phenotypic values of the BnaZN-RIL population showed normal or near-normal distribution for all 21 traits across the six environments (Additional file 1: Figure S1). Analysis of variance showed that genotype, environment, and their interaction had significant effects on all 21 investigated traits (Additional file 9: Table S2).

**Fig. 2:**
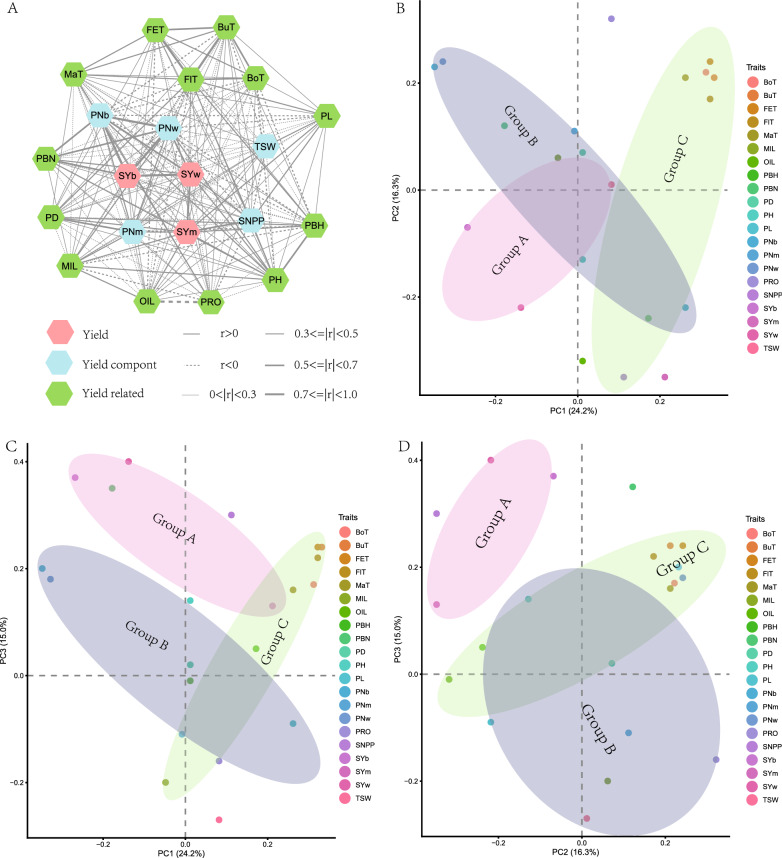
Trait relationship revealed by trait network and PCA analysis of 21 traits investigated in the BnaZN-RIL population. **A:** Trait network constructed by the 149 trait pairs showing significant correlation in at least half of the investigated environments. The seed yield and its components and related traits are shown in different colors. The traits are treated as nodes, which are linked with liens of different types (solid: + ; dotted: -) and widths (distinguishing the degree). **B**–**D:** Loading of PC1 vs PC2, PC1 vs PC3, and PC2 vs PC3. The different traits are represented by dots of different colors as demonstrated in the legends. Circle **A**, **B,** and **C** represent the different groups

Among the 210 trait pairs, 198 (94.3%) showed a significant correlation in at least one environment and 158 (75.2%) were significantly correlated in multiple environments (Additional file 2: Figure S2; Additional file 10: Table S3). To obtain a general picture of the trait relationship, a trait network map was constructed using the correlation that was significant in more than half of the investigated environments (Fig. 2A), which displayed several obvious characteristics. First, all 21 investigated traits were woven into a complex network of interconnections, and none was independent. Second, seed yield was located at the center of this network, followed by yield components, whereas other yield-related traits were on the periphery. Third, seed yield showed a higher correlation with yield components than with other yield-related traits. In addition, yield-related traits usually showed a higher correlation with yield components than with yield itself, suggesting their indirect relationship with seed yield.

Although the results of principal component analysis (PCA) in Wuhan and Zhengzhou showed a slight difference, high consistency was found between different years at the same location (Additional file 11: Table S4). Therefore, the best linear unbiased prediction (BLUP) value for six environments was subjected to PCA (Table 1; Fig. 2B–D). The first principal component 1 (PC1) accounted for 24.2% of the trait variance. Among the seven traits (pod number of branch raceme (PNb), pod number of whole plant (PNw), seed yield of branch raceme (SYb), primary branch number (PBN), seed yield of whole plant (SYw), main inflorescence length (MIL), pod number of main raceme (PNm)) with negative loading, PNb and PNw showed a high value, which suggested that seed yield was primarily determined by pod number. Of the other 14 traits with positive loading, five growth period traits (budding time (BuT), flowering time (FlT), flowering end time (FET), bolting time (BoT), maturity time (MaT)) had the highest value. The second principal component (PC2) accounted for 16.3% of the trait variance. Among the eight traits with negative loading, the seed yield of the main raceme (SYm), seed number per pod (SNPP), and seed oil content (OIL) showed the highest values, which indicated that seed yield was secondarily determined by seed number per pod. Of the other 13 traits with positive loading, PRO, FET, PNw, and PNb had a high value, which was in accordance with the significant negative correlation between seed number per pod and pod number as well as between oil content and protein content (Additional file 2: Figure S2; Additional file 10: Table S3). The third principal component (PC3) accounted for 15.0% of the total variance. Of the six traits with negative loading, thousand-seed weight (TSW), MIL, and PRO showed a high value, which was in accordance with a positive correlation between seed weight and protein content in the current study (Additional file 2: Figure S2; Additional file 10: Table S3) and previous research [34]. Of the other 15 traits with positive loading, SYw, SYb, PBN, and SNPP exhibited a high value, which was in accordance with the negative correlation between seed weight and seed number per pod, branch number, and seed yield (Additional file 2: Figure S2; Additional file 10: Table S3).

**Table 1:** Principal component analysis of the 21 traits based on the BLUE (best linear unbiased estimation) value in six environments

Trait name (abbreviation)	PC1	PC2	PC3
Bolting time (BoT)	0.312	0.223	0.172
Budding time (BuT)	0.334	0.208	0.242
Flowering ending time (FET)	0.315	0.238	0.238
Flowering time (FlT)	0.323	0.168	0.222
Maturity time (MaT)	0.265	0.209	0.157
Main inflorescence length (MIL)	− 0.048	0.065	− 0.195
Oil content of seeds (OIL)	0.014	− 0.318	− 0.011
Primary branch height (PBH)	0.166	− 0.244	0.054
Primary branch number (PBN)	− 0.181	0.122	0.355
Pod density (PD)	0.013	0.075	0.018
Plant height (PH)	0.013	− 0.132	0.139
Pod length (PL)	0.256	− 0.220	− 0.092
Pod number of branches (PNb)	− 0.348	0.232	0.201
Pod number of main raceme (PNm)	− 0.013	0.106	− 0.108
Pod number of whole plant (PNw)	− 0.326	0.238	0.181
Protein content of seeds (PRO)	0.082	0.323	− 0.156
Seed number per pod (SNPP)	0.112	− 0.349	0.302
Seed yield of branches (SYb)	− 0.265	− 0.065	0.367
Seed yield of main raceme (SYm)	0.209	− 0.355	0.129
Seed yield of whole plant (SYw)	− 0.141	− 0.223	0.399
Thousand seed weight (TSW)	0.084	0.012	− 0.273
Eigenvalue	5.082	3.423	3.145
Proportion of variance	0.242	0.163	0.150
Cumulative proportion	0.242	0.405	0.555

### Analysis of a high-density genetic map revealed a concentrated distribution of distorted segregation, residual heterozygosity, and variation in recombination frequency

**Fig. 3:**
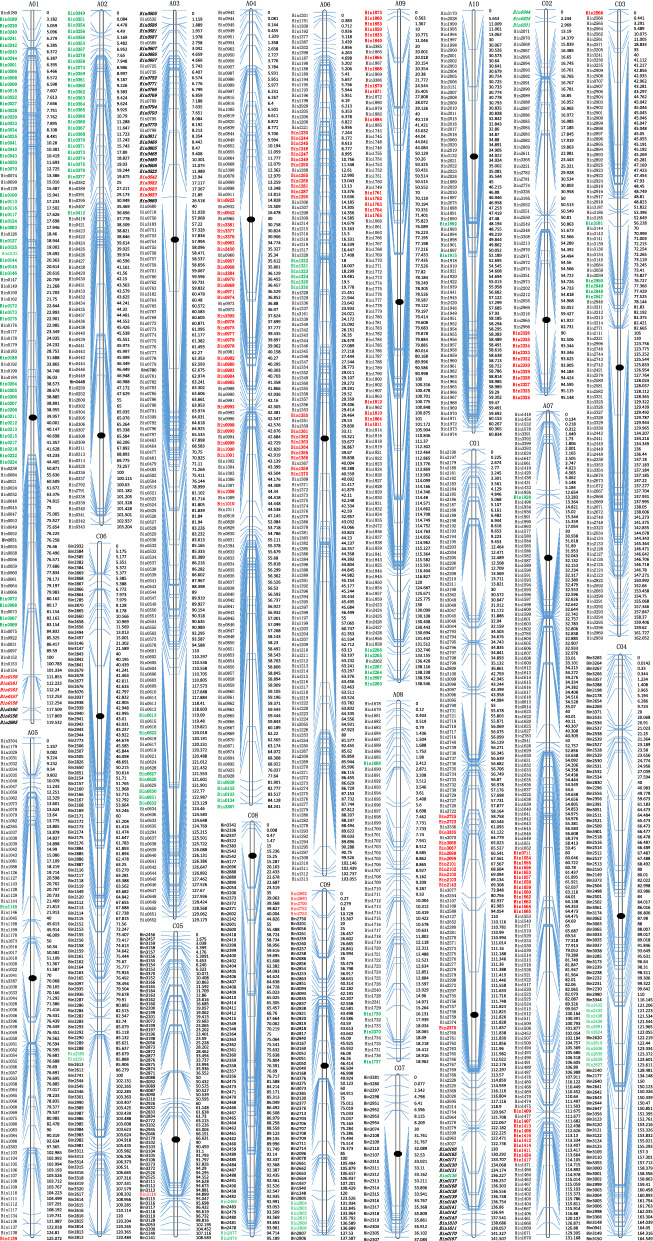
Genetic linkage map constructed using the BnaZN-RIL population. The names of each of the 19 linkage groups are shown on the top and the black oval indicates the positions of the centromere. The serial numbers of mapped bins and their genetic distances are shown on the left and right sides of each linkage group, respectively. The names of bin markers with high heterozygosity and segregation distortion are highlighted using italics and color, of which red and green distinguish the skew from Zhongshuang11 and No.73290, respectively

To further dissect the genetic relationship between yield traits, a high-density genetic linkage map of 2207.7 cM and 1887 bins/6444 SNP markers was constructed for the BnaZN-RIL population, which covered 812.1 Mb physical distance representing 84.5% of the assembled genome of Zhongshuang11 (Fig. 3; Table 2). It should be noted that the recombination frequencies (2.92 to 5.93) of the 10 linkage groups in the A subgenome were all higher than those (1.95 to 2.75) for the 9 linkage groups in the C subgenome, with a mean of 2.72 per Mb.

**Table 2:** The summary statistics of the BnaZN-RIL linkage map

Linkage group	No. of Bin/SNP	Genetic distance	Physical distance	Recombination frequency^a^	No. of marker skewed to			Closet marker covering centromere		
					ZS11	73,290	Total	Physic distance	Genetic distance	Recombination frequency
A01	106/307	139.5	37.2	3.75	5	57	62	11.66	1.90	0.16
A02	84/190	103.2	31.6	3.27	0	29	29	16.81	17.53	1.04
A03	143/293	129.2	27.6	4.68	4	7	11	/	/	/
A04	131/264	84.2	25.3	3.33	35	5	40	7.80	5.28	0.68
A05	83/261	126.0	40.5	3.11	2	0	2	16.27	0.25	0.02
A06	160/314	152.1	48.4	3.14	21	6	27	29.26	8.49	0.29
A07	163/451	135.0	31.8	4.24	32	1	33	6.43	0.27	0.04
A08	61/106	19.0	3.2	5.93	0	4	4	/	/	/
A09	112/274	188.5	64.6	2.92	20	6	26	33.20	11.85	0.36
A10	77/139	64.8	19.4	3.34	0	2	2	10.57	0.78	0.07
C01	142/385	141.5	56.2	2.52	13	1	14	15.11	1.75	0.12
C02	59/192	125.1	63.5	1.97	11	3	14	21.19	5.64	0.27
C03	107/664	162.1	75.2	2.16	1	5	6	5.84	0.00	0.00
C04	105/1685	164.5	70.6	2.33	0	11	11	5.83	0.00	0.00
C05	71/215	108.6	55.6	1.95	1	0	1	5.42	0.00	0.00
C06	121/508	120.5	43.9	2.75	0	2	2	/	/	/
C07	24/82	31.1	12.0	2.60	0	0	0	14.05	5.69	0.40
C08	71/289	95.2	45.5	2.09	0	3	3	14.17	1.76	0.12
C09	67/329	117.6	60.1	1.96	5	6	11	10.55	0.96	0.09
Total	1887/6444	2207.7	812.1	2.72	150	148	298	224	62.13	0.28

^a^Recombination frequency is calculated as the genetic distance (cM) divided by physical distance (Mb)

^b^Marker density is calculated as number of Bin/SNP markers divided by physical distance (Mb)

^c^Coverage ratio is calculated as the covered physical distance (Mb) of linkage groups divided by the length (Mb) of the assembled pseudochromosomes

Notably, 298 bins (15.8%) displayed extremely significant segregation distortion, which tended to cluster especially at the end of linkage groups (Fig. 3; Table 2). Interestingly, dozens of markers were found to have high residual heterozygosity (ranging from 25.6 to 60.2%) in the BnaZN-RIL linkage map, most of which were concentrated at the ends of A01, A03, C01, and C02. Further single marker analysis showed that many of these markers in linkage groups A03 and C02 displayed an overdominant effect on pod number, the most important principal component of seed yield (Additional file 12: Table S5).

**Fig. 4:**
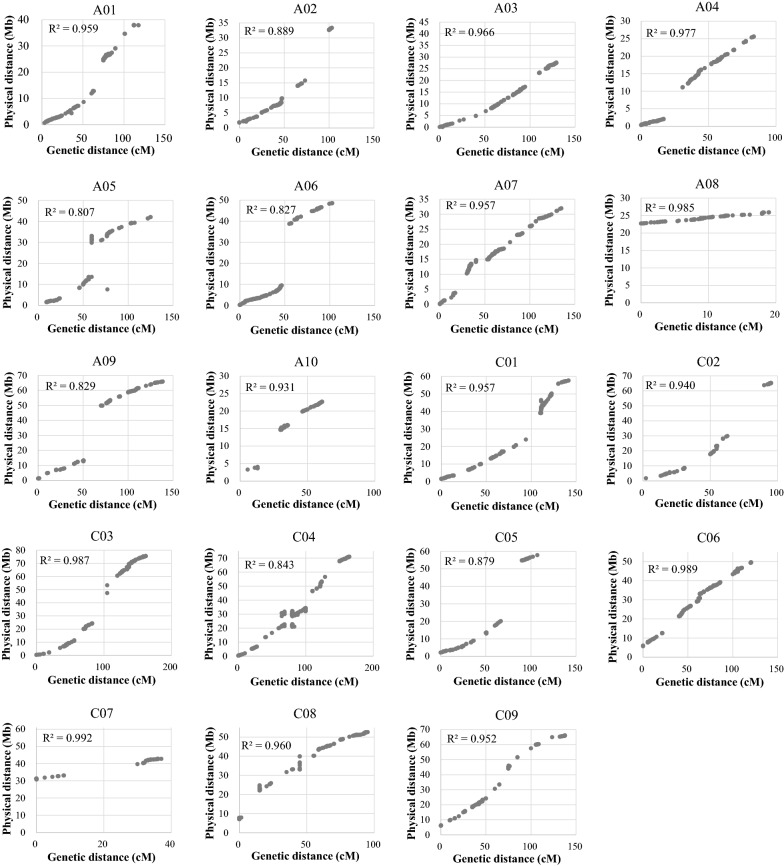
Alignment of the genetic map of the BnaZN-RIL population and the genomic map of Zhongshuang11. The horizontal and vertical axes show the genetic distances (cM) and physical positions (Mb), respectively. The scatter dots indicate the genetic positions of the mapped SNP markers on the genetic map of the BnaZN-RIL population and their physical positions on the pseudochromosomes (A01-A10; C01-C09) of the reference genome Zhongshuang11. The determination coefficients of the genetic and physical distances of these SNP markers are also shown on each chart

The constructed BnaZN-RIL genetic linkage map aligned well with the genomic map of Zhongshuang11 (Fig. 4), demonstrating its high quality. Generally, the genetic distance increased with physical distance, with a more rapid increase at both ends of the chromosomes than in the middle part, showing an S-shaped curve. There were obvious breakpoints (no markers in a large genome segment) in the alignment map, basically corresponding to the centromeric region. Interestingly, no SNP marker was located in the centromeric region, and markers flanking centromeric regions were basically monomorphic (Additional file 13: Table S6). The recombination frequencies in pericentromeric regions ranged from 0.02 (A05) to 1.04 (A02) with a mean of 0.28 per Mb, which was much lower than the corresponding mean (2.72 per Mb) calculated from the whole genome.

### Analysis of genetic relationships revealed an integrated trait-QTL network with hub-QTL to control yield

To further dissect the genetic determination of yield, large-scale QTL mapping was conducted using the abovementioned phenotypic data and high-density linkage map of the BnaZN-RIL population. At the significance level of *P* = 0.05, a total of 207 QTL were identified for the 21 traits investigated in six environments, which explained 4.0–48.3% of the variance (Additional file 14: Table S7A). After the integration of overlapping identified QTL trait by trait in different environments, a total of 134 consensus QTL were obtained (Additional file 14: Table S7B). Of these, 19, 32, and 84 were for seed yield, yield component, and related traits, respectively.

**Fig. 5:**
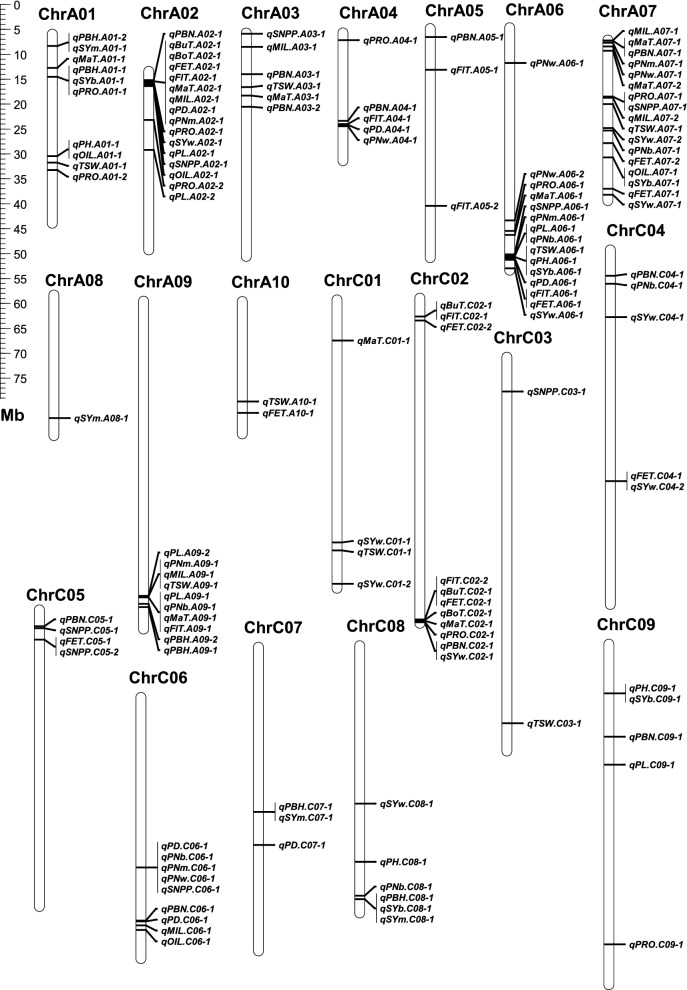
Distribution of consensus QTL in the Zhongshuang11 genome. The 19 chromosomes are drawn as cylinders, with their names (A01-A10 and C01-C09) shown on the top. The positions of each QTL are indicated by thin horizontal lines

Interestingly, the consensus QTL were clustered rather than randomly distributed across the genome (Fig. 5), which might explain the extensive correlation among these traits (Additional file 2: Figure S2). Then, 106 of the 134 consensus QTL were combined into 28 QTL clusters (Additional file 14: Table S7C), which might be caused by pleiotropy or tight linkage. Among the 19 consensus QTL for seed yield, 16 overlapped with the QTL for other traits (Additional file 14: Table S7C), highly in accordance with the extensive correlation between yield and other investigated traits (Additional file 2: Figure S2; Additional file 10: Table S3). Statistical analysis of the number/proportion and direction of these overlapping QTL revealed some obvious characteristics (Additional file 15: Table S8). First, the directions of these overlapping QTL between seed yield and other yield components or related traits were same rather than opposite. Second, the directions of almost all overlapping QTL of the five growth period traits were same, indicating that pleiotropy rather than tight linkage was more likely to be the underlying genetic basis.

It should be noted that four QTL clusters (QC4, QC8, QC14, QC17) contained many consensus QTL with large effects (Additional file 14: Table S7C), which might play an important role in regulating traits. Of the 14 QTL involved in QC4 at the top of chromosome A2, eight showed reproducible and large effects, including three growth period traits (+ , 25.4–48.3%), seed yield (+ , 10.9%), PN (+ , 23.3%), PBN (+ , 13.0%), protein (−, 18.8%) and oil content (+ , 11.1%). QC8 on the lower part of chromosome A06 showed reproducible and large effects on SNPP (+ , 25.4%), PN (−, 17.7%), and pod length (PL) (+ , 8.8%), but a moderate effect on other traits including seed weight (−, 9.9%), pod density (PD) (−, 9.1%), FIT (−, 6.5%), and plant height (PH) (−, 12.4%). QC14 on the lower part of chromosome A09 showed reproducible and large effects on PL (+ , 18.8%) and seed weight (+ , 11.9%), and moderate effects on six other traits including PN (−, 8.2%), FIT (−, 4.1%), MaT (−, 13.5%), main stem length (MIL)(−, 7.0%), and primary branch height (PBH) (−, 11.0%). QC17 on the bottom of chromosome C02 had producible and large effects on growth period traits (−, 7.0%−16.4%), with a moderate effect on seed yield (−, 8.3%), PBN (−, 6.0%), and protein content (−, 6.4%).

**Fig. 6:**
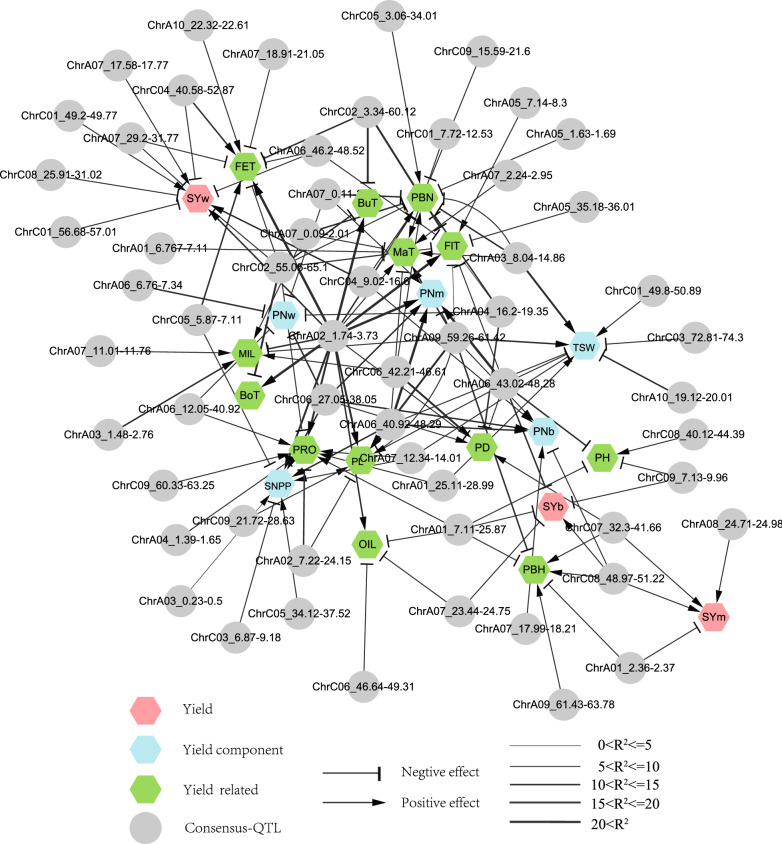
Trait–QTL network constructed for all of the investigated traits and the underlying consensus QTL. Traits and consensus QTL are treated as nodes, which are drawn using the hexagon and circle, respectively. The seed yield, yield components and yield-related traits are distinguished by the different colors. The additive-effect direction of these consensus QTL is distinguished by the lines of different endpoint types (+ : arrow; -: vertical line). The abbreviations of trait names are the same as those in Table 1, and the width of the lines indicates the R^2^ of the consensus QTL

To further link the phenotypic and genetic relationship between yield and its components or related traits, an integrated trait–QTL network was constructed (Fig. 6), which displayed several obvious features. Firstly, all 134 consensus QTL for the 21 traits were integrated into an interconnected network, none of which was independent. This indicated the extensive relationships between these traits, which might be caused by the multiple/pleiotropic roles of the underlying QTL. Secondly, there were several obvious hub-QTL that were linked with multiple traits and displayed large effects, which might play a major role in the trait–QTL network and are worthy of further study. Thirdly, most of these hub-QTL clusters had smaller effects on seed yield than their components or related traits, which indicated their pleiotropy and indirect effects on yield.

### Further dissection of four representative hub-QTL revealed the causal trait relationship and underlying target or candidate genes in yield determination

To further dissect the complex trait relationship within yield determination at molecular level, four representative hub-QTL were selected to construct high-generation near isogenic lines (NILs) (Additional file 3: Figure S3) for accurate evaluation of their phenotypic effects and trait relationships as well as fine-mapping and identification of underlying genes.

### *BnaA2.FLC* indirectly affected yield through influencing three yield components via regulating the growth period

**Fig. 7:**
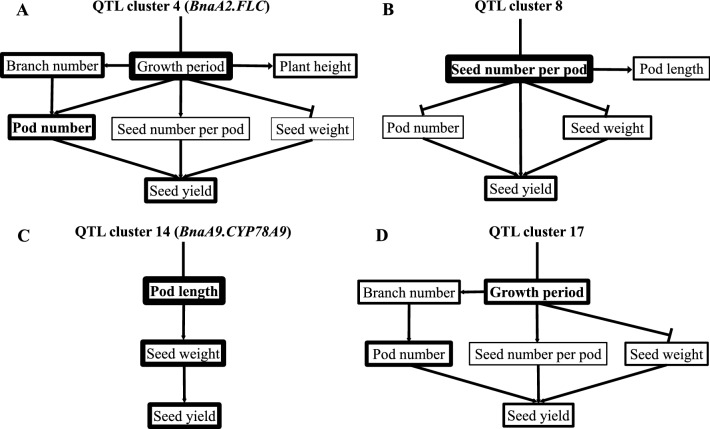
Further dissection of four representative hub-QTL clusters (**A**–**D**: QC4, QC8, QC14, QC17). The up-/downstream and positive/negative feedback relationships between the relevant traits were linked with lines of different widths and types, which indicate the size and direction (positive: arrow; negative: vertical line) of the effect, respectively

QC4 was narrowed to a 71-kb region between SNP markers seq-new-rs24859 (A02: 1,971 kb) and seq-new-rs32262 (A02: 2,043 kb). Relative to Zhongshuang11 (Table 3), the homologous NIL_QC4 showed the largest decrease in BoT (− 26.1%), followed by BuT, SYb, PNb, SYw, PBH, PNw, and PBN (from − 19.8 to − 14.2%), while SYm, PNm, FlT, PH, and PD showed a moderate reduction (from − 12.0 to − 8.9%), whereas the other eight traits showed only small change (from − 5.0 to 6.2%). These results were generally consistent with their effects on these traits in the preliminary mapping population of BnaZN-RIL (Additional file 14: Table S7C), such as the largest effect on several growth period traits. Further conditional QTL analysis using the NIL segregation population demonstrated a complex up-/down-stream and positive/negative regulation between these traits (Fig. 7A), i.e., QC4 had a large and direct effect on the growth period, which then had indirect and pleiotropic effects on PH ( +), PBN ( +), PN ( +), SNPP ( +), seed weight (−), and the final seed yield ( +). This was understandable because longer vegetative growth generally produces more leaves and biomass, therefore positively correlating with branch and pod number [35]. In the semi-winter growing area of China, oilseed rape cultivars with late maturity often encounter high-temperature ripening, leading to the decreased seed weight due to inadequate seed filling [36].

**Table 3:** Phenotypic evaluation of the recurrent parent Zhongshuang11 and corresponding NILs for four representative hub-QTL clusters

Trait	Zhongshuang11	NIL_QC4	NIL_QC8	NIL_QC14	NIL_QC17
SYw	17.3 ± 1.71	14.5 ± 1.45	15.1 ± 1.57	14.2 ± 1.31	19.1 ± 1.83
SYb	11.28 ± 1.04	9.23 ± 0.8	9.77 ± 0.86	9.41 ± 0.81	12.7 ± 1.13
SYm	6.01 ± 0.51	5.29 ± 0.51	5.31 ± 0.48	4.83 ± 0.46	6.36 ± 0.57
PNw	210 ± 19.7	180 ± 18.1	222 ± 21.7	216 ± 20.1	237 ± 22.1
PNb	142 ± 15	119 ± 13.2	149 ± 15.8	147 ± 15.6	163 ± 16.1
PNm	68.5 ± 6.42	61.4 ± 5.73	73.2 ± 7.3	69.3 ± 6.81	74.4 ± 7.43
SNPP	21.1 ± 0.91	20.0 ± 0.88	16.1 ± 0.84	20.7 ± 0.9	22.1 ± 0.93
TSW	4.16 ± 0.19	4.29 ± 0.2	4.51 ± 0.21	3.37 ± 0.17	3.87 ± 0.18
BoT	128 ± 1.54	94.7 ± 0.71	128 ± 1	128 ± 1	148 ± 2.12
BuT	146 ± 2.24	117 ± 1.71	146 ± 2	146 ± 2	165 ± 2.66
FlT	165 ± 1.24	149 ± 0.71	166 ± 1.41	165 ± 1.15	177 ± 2.12
FET	187 ± 1.24	180 ± 0.71	188 ± 1	187 ± 1.41	192 ± 1.83
MaT	219 ± 0.71	215 ± 1	219 ± 0.86	218 ± 1	221 ± 0.93
PH	176 ± 9.92	159 ± 8.44	177 ± 10.05	175 ± 9.31	182 ± 10.15
PBH	86.7 ± 6.73	73.8 ± 5.22	87.8 ± 8	85.6 ± 6.59	90.0 ± 8.18
PBN	5.13 ± 0.64	4.40 ± 0.51	5.36 ± 0.71	4.98 ± 0.63	5.59 ± 0.76
MIL	57.1 ± 4.42	56.2 ± 4.19	58.3 ± 4.71	58.12 ± 4.54	58.9 ± 4.84
PD	1.20 ± 0.13	1.09 ± 0.1	1.26 ± 0.15	1.19 ± 0.11	1.26 ± 0.17
PL	86.7 ± 5.41	83.2 ± 4.96	80.4 ± 4.46	64.1 ± 3.26	89.0 ± 5.42
OIL	48.3 ± 2.48	47.7 ± 2.13	48.1 ± 2.3	48.6 ± 2.64	48.7 ± 2.84
PRO	17.8 ± 1.48	18.9 ± 1.89	18 ± 1.66	17.4 ± 1.34	17.1 ± 1.19

The abbreviations of the trait names are the same as those in Table 1

The fine-mapped region of QC4 contained only 17 annotated genes in the reference genome of Zhongshuang11 (Table S9), only BnaA02G0035100ZS(*BnaA2.FLC*) was homologous to the known flowering time gene *FLC* (AT5G10140) in *Arabidopsis*. *BnaA2.FLC* had been previously identified to be the causal [37] or candidate [38] gene for flowering time major QTL in the same genomic region of chromosome A2. In addition, RNA sequencing of the shoot apical meristem at the initial stage of floral bud differentiation showed that the expression of *BnaA2.FLC* was much higher (56.8-fold) in Zhongshuang11 than in No.73290 (Additional file 17: Table S10), which was highly in accordance with the positive additive-effect direction of QC4 on growth period traits (Additional file 14: Table S7). Further sequence analysis showed that there was a 10-bp insertion in the core 40-bp motif of the promoter in No.73290 (Additional file 4: Figure S4), which might decrease its expression. These results highly supported that *BnaA2.FLC* was the target gene of QC4.

### QC8 directly affected yield by regulating seed number per pod likely through the embryogenesis gene *BnaA6.EMB93*

QC8 had previously been fine-mapped to a 267-kb region between SSR markers BrSF47-10 and BrSF46-167, using the BC_4_F_2_ population and its recombinant progeny [39]. However, no recombinant was found in a small genomic fragment of 103 kb between SSR markers BrSF46-28 and BrSF46-78, even in a very large BC_5_F_2_ population of 37,976 plants [40]. Relative to Zhongshuang11 (Table 3), the homologous NIL_QC8 showed the largest decrease in SNPP (− 23.8%), followed by SYb (− 13.4%), SYw (− 12.8%), and SYm (− 11.6%), while TSW (+ 8.4%), PL (− 7.3%), PNm (+ 6.9%), PNw (+ 5.7%), and PNb (+ 5.1%) showed moderate change, and the differences of other 12 traits were not significant. These results were also highly consistent with their effects on these traits in the preliminary mapping population of BnaZN-RIL (Additional file 14: Table S7C), such as the largest effect on seed number per pod. Conditional QTL analysis using the NIL segregation population also revealed the causal relationships between these traits (Fig. 7B), where QC8 showed a direct and large effect on seed number per pod, which then had indirect and pleiotropic effects on PL ( +), PN (−), and seed weight (−). These results were highly consistent with the previous finding that the change in seed number per pod is usually accompanied by a change in fruit length in the same direction (but the reverse is not true), indicating positive feedback between seed setting and fruit growth [41]. In addition, the opposite pleiotropy between SNPP and pod number/seed weight could be explained by the competition among sink organs due to limited resources, which resulted in trade-off/negative feedback between them [39]. However, the moderate negative effects of QC8 on pod number and seed weight could not counteract its large positive effect on seed number per pod. It also had a considerable positive effect on the final seed yield.

Among the 19 genes annotated in the 103-kb region of QC8 (Additional file 16: Table S9), only BnaA06G0400200ZS’s homologue (*EMB93*/*AT2G03050*) is involved in known biological processes (such as ovule differentiation and development, fertilization and seed development) related to seed number in *Arabidopsis*. The *EMB93* gene encodes a mitochondrial transcription termination factor that is involved in embryogenesis, whose mutation results in embryo lethality (https://www.arabidopsis.org/servlets/TairObject?id=34053&type=locus). There were 18 SNPs between the coding sequences of *BnaA6*.*EMB93* in Zhongshuang11 and No.73290, only one of them caused amino acid variation that was not in the functional domains (Additional file 5: Figure S5). However, there was an ≈11 kb insertion in the upstream regulatory region of *BnaA6*.*EMB93* in No.73290 but not in Zhongshuang11, which was highly consistent with its decreased expression in the ovules of different stages in NIL_QC8 (Additional file 6: Figure S6). These results suggested that *BnaA6*.*EMB93* was the most likely candidate gene of QC18.

### The cytochrome p450 gene *BnaA9.CYP78A9* indirectly affected yield through influencing seed weight via regulating pod length and photosynthetic area.

QC14 was successfully delimited to a 90-kb region between SNP markers Bn-A09-p30171993 (A09: 57,344 kb) and Bn-A09-p30260475 (A09: 57,435 kb). Compared to the recurrent parent Zhongshuang11, the homologous NIL_QC14 showed the largest decrease in PL (− 26.1%), followed by SYm (− 19.6%), TSW (− 19.0%), SYw (− 17.6%), and SYb (− 16.6%), whereas the other two yield components and 14 yield-related traits exhibited no significant difference (Table 3). The effects of QC14 in high-generation NILs were highly similar to those in the preliminary mapping population of BnaZN-RIL (Additional file 14: Table S7C), where it had a stable and large effect on pod length, followed by seed weight. Conditional QTL analysis further revealed the causal relationship between these traits (Fig. 7C), where QC14 had a direct effect on pod length, thus had indirect pleiotropic effects on seed weight ( +), and final yield ( +). This is understandable as longer pod generally means a larger photosynthetic area that is able to produce more assimilates for seed filling [34], which essentially reflects the positive feedback between the source and sink.

Among the 13 genes annotated in the 90-kb region of QC14 (Additional file 16: Table S9), only BnaA09G0560100ZS’s homologue (*CYP78A9*/AT3G61880) is involved in regulating silique and seed development in *Arabidopsis* (https://www.arabidopsis.org/servlets/TairObject?id=36508&type=locus). In addition, the expression level of BnaA09G0560100ZS showed a large difference (fold-change = 24.0 and 3.3; *P*-value = 1.2E−13 and 8.7E−3) between Zhongshuang11 and NIL_QC14 in both pod walls and seeds (Additional file 6: Figure S6). Although the coding sequence of BnaA09G0560100ZS had no difference between Zhongshuang11 and NIL_QC14, a CACTA-like transposable element was present in its upstream regulatory region in Zhongshuang11, but absent in NIL_QC14 (Additional file 7: Figure S7). A very recent study showed that this CACTA-like transposable element in the upstream region of *BnaA9.CYP78A9* acted as an enhancer to increase its expression, which was responsible for a major QTL-*qSLW.A9* for silique length and seed weight in rapeseed [42]. All of the above results strongly supported that *BnaA9.CYP78A9* was the target gene of QC14.

### QC17 indirectly affected yield by influencing three yield components likely through the growth period gene *BnaC2.MAF2*

QC17 was narrowed to a 744 kb genomic region between two SNP markers Bn-scaff_16139_1-p1393867 and seq-new-rs22829. Compared to the recurrent parent Zhongshuang11 (Table 3), the homologous NIL_QC17 showed the largest increase on BoT (+ 15.5%) and PNb (+ 15.1%), followed by PNw (+ 13.0%), BuT (+ 12.8%), SYb (+ 12.6%), SYw (+ 10.2%), PBN (+ 9.0%), and PNm (+ 8.6%), and moderate changes in FlT (+ 7.2%), TSW (− 7.0%), SYm (+ 5.9%), PD (+ 5.3%), and SNPP (+ 4.7%). The effects of QC17 on these traits were similar to those in the preliminary mapping population of BnaZN-RIL (Additional file 14: Table S7), where it had stable and large effects on growth period traits. The integration with conditional QTL results demonstrated the causal relationship between traits controlled by QC17 (Fig. 7D), where it had a direct and large effect on the growth period, which then had indirect and pleiotropic effects on PBN ( +), PN ( +), SNPP ( +), seed weight (−), and the final yield ( +).

The detailed analysis of the 101 genes annotated in the 744 kb region of QC17 revealed seven genes (BnaC02G0541300ZS, BnaC02G0542400ZS, BnaC02G0542800ZS, BnaC02G0545600ZS, BnaC02T0546100ZS, BnaC02T0546200ZS, and BnaC02T0546300ZS) that are homologous to the known genes (*LNK*, *MTL1*, *EIP9*, *MAF2*, and *MAF4*) controlling flowering time in *Arabidopsis* (Additional file 16: Table S9). Notably, all seven genes showed no expression or very low expression levels except for *BnaC2.MAF2* (Additional file 17: Table S10), which was the most likely candidate gene of QC17.

## Conclusions

In the current study, yield was systematically dissected at the phenotypic, genetic, and molecular levels using oilseed rape as an example. At the phenotypic level, analysis of 21 traits in a representative recombinant inbred line (RIL) population showed that yield was determined by a complex trait network with key contributors. At the genetic level, large-scale mapping and analysis of QTL showed that yield was controlled by an integrated QTL network with obvious hub-QTL that regulated multiple traits with large effects. At the molecular level, four representative hub-QTL were further fine-mapped, the causal relationships between the relevant traits were revealed, and the target or candidate genes were also identified. The highly consistent results at the phenotypic, genetic, and molecular dissecting provided a systematic view and further insight into the determination of yield in crops.

## Discussion

### Yield was determined by a complex trait network with key contributors

In the current study, there were several important findings in dissecting the phenotypic relationship between yield and its components or related traits by using the different analysis methods. Additionally, the constructed trait network exhibited an obvious center-periphery structure, where seed yield was located at the center, followed by yield components and related traits (Fig. 2A). This suggested that yield-related traits could indirectly affect yield by influencing yield components, which was highly supported by the results of further dissection of four representative hub-QTL. Among them, QC 8 directly affected yield, whereas QC4, QC14, and QC17 indirectly influenced yield. Further principal component analysis revealed several key traits (mainly represented by three yield components) as well as their influencing factors in determining yield. For example, seed yield was mainly determined by pod number and branch number, which were largely influenced by growth period, which was also highly supported by the results of further dissection of QC4 and QC17 (Fig. 7A, D). These results were highly accordant with population-level studies showing that the high yield of oilseed rape mainly depended on more branch number and pod number, followed by more and larger seeds [35, 43, 44].

### Analysis of an integrated genetic and physical map provided insight into evolution and heterosis

The in-depth analysis of the integrated genetic and physical map resulted in several novel/interesting findings, which had great significance for genetics and breeding. To our knowledge, this is the first report that has calculated the recombination frequency in centromeric regions and compared with other regions in *Brassica*, although the genome-wide recombination frequency has been estimated [45]. The higher recombination frequencies of chromosomes A1 to A10 than C1 to C9 (Table 2) might be attributed to the lower proportion of repetitive sequences in the A subgenome than in the C subgenome [46, 47]. The probability of segregation distortion showed a gradual downward trend from the peak marker with the most significant Chi-square value (P_χ_^2^), which might be closely linked with the segregation distortion loci [48]. Interestingly, all three major QTL clusters of the growth period were distorted to alleles with early growth/development (Fig. 3; Additional file 14: Table S7), which might be subjected to selection during the development of the BnaZN-RIL population. This result strongly suggested that selection via a genetic hitchhiking effect had an important role in the generation of segregation distortion in this population [49]. The higher proportion of distorted markers in the current BnaZN-RIL population than in the previously reported BnaZN-F2 population [45] derived from the same parents could be largely due to more generations of selection in the RIL population than in the F_2_ population [50]. Unexpectedly, several regions with high (even > 50%) residual heterozygosity were found in the BnaZN-RIL population that had been self-crossed for seven generations (theoretical heterozygosity was only 1.56%). Interestingly, the markers with high heterozygosity tend to cluster at the end of linkage groups (Fig. 3), and the heterozygotes in these regions usually perform better than the corresponding homozygotes (Additional file 12: Table S5), which has great significance for evolution and heterosis [51].

### Yield was controlled by an integrated trait-QTL network with obvious hub-QTL

The integrated high-quality genetic and physical map provided an ideal platform to accurately dissect the genetic relationship between yield and its components or related traits. The most significant characteristics regarding these QTL were their clustered distribution (Fig. 5) and high-proportion (> 80%) of overlap (Additional file 15: Table S8), which were highly consistent with the extensive correlation between these traits (Additional file 3: Figure S3). The extensive trait correlation and QTL overlap in the current study were further supported by an integrated trait-QTL network that involved all of the 134 consensus QTL for 21 traits (Fig. 6). This strongly indicated the complexity of the genetic improvement of composite traits such as yield [1]. Generally, the overlapping QTL clusters/hotspots of yield [18–20, 29, 52–54] usually include several other QTL for yield components and/or related traits. Therefore, the overlapping QTL cluster for yield can be caused by the pleiotropic effects of a specific gene or the combined effects of several tightly linked genes, since a detected QTL in preliminary mapping may be dissected into several sub-QTL after further fine-mapping [24, 55, 56]. It is speculated that some of the seed yield QTL could not be detected due to the cancellation of opposite effects of several tightly linked QTL for the same or different yield components or related traits. Relative to yield components or related traits with higher heritability, yield QTL were generally unrepeatable and showed smaller effects, which made it difficult to directly clone and utilize them. The integrated trait-QTL network showed obvious hub-QTL clusters that linked multiple traits and displayed large effects, which played a major role in trait relations and were likely to be the targets for cloning.

### Hub-QTL dissection provided causal explanations for yield formation and determination

Through the further dissection of four representative hub-QTL clusters using high-generation NILs, the qualitative (up/down-stream and positive/negative regulation) and quantitative (effect size) relationships between the relevant yield traits were revealed (Fig. 7). To our knowledge, this should be the exact roadmap of yield determination at the single locus/gene level, which provided a further causal explanation for yield formation. First, there were two main pathways for yield formation, including direct determination by yield components such as seed number per pod (QC8) and indirect determination by yield-related traits, such as growth period (QC4 and QC17) and source size (QC14). Second, if a yield QTL was directly caused by the genes of a specific yield component, it generally had negative pleiotropy on other yield components. Highly consistent with this, none of the cloned genes for yield QTL can simultaneously improve all yield components [42, 57–67]. This universal rule can explain the common phenomenon for QTL clusters of yield traits in which their effects on yield are usually smaller than those on yield components. However, the size of the effect was related to the determination period/developmental order of these traits (i.e., pod number sooner than seed number per pod sooner than seed weight), the traits determined earlier usually have large influence on those determined later but not vice versa. Taking QC8 as an example, the change in seed number per pod usually causes a large change in seed weight but only a small change in pod number. In addition, the trait relationships reflected by the four major hub-QTL clusters demonstrated that the yield variation in the BnaZN-RIL population should be mainly attributable to the difference in source (QC4/QC17 and QC14 affect biomass accumulation and pod area, respectively) and sink (QC8 controls ovule fertility and seed number per pod). The results of further dissection at the molecular level were also highly consistent with those at the genetic and phenotypic levels (e.g., pleiotropy of *BnaA9.CYP78A9* → QTL cluster 14 → correlation between pod length and seed weight), which provided a systematic and deep understanding of yield determination.

## Experimental procedures

### Population development

The BnaZN-RIL population was developed by single-seed descent from the previously reported BnaZN-F_2_ population that was derived from Zhongshuang11 and No.73290 [45]. For the development of the near-isogenic line, the F1 hybrid (Zhongshuang11 × No.73290) was crossed with Zhongshuang11 for 12 generations. For each generation, BCnF_1_ plants heterozygous at the target QTL were screened (using flanking SSR/InDel markers) for each consecutive backcross. For the final BC_12_F_1_ generation, the heterozygous plants were also screened by background selection (using Brassica 60 K Illumina^®^ Infinium SNP array), of which those with the highest background recovery rate (99.6%, 99.7%, 99.8%, and 99.5% for QC4, QC8, QC14, and QC17, respectively) were self-crossed to produce BC_12_F_2_ seeds.

### Field experiments

Both the BnaZN-RIL population and its parents were planted in six environments, including four years at Wuhan (codes W12, W13, W14, and W16) and two years at Zhengzhou (codes Z13 and Z14). To accurately evaluate the phenotypic effect of four hub-QTL clusters, Zhongshuang11 and the corresponding NILs were planted and investigated at Wuhan in 2019. The field planting followed a randomized complete block design with three (BnaZN-RIL population) or ten (NILs) replications, respectively. Each block contained three (BnaZN-RIL population) or five (NILs) rows, respectively, with 33-cm spacing and 18 plants were evenly retained after singling. At maturity, 10 (BnaZN-RIL population) or 30 (NILs) representative individuals in the middle of each block (except for two rows on the side) were harvested from each block.

### Trait investigation

A total of 21 traits were investigated and divided into five types: seed yield, yield components, growth period, plant architecture and seed constituent. Pod number (PN), seed number per pod (SNPP), pod length (PL),thousand-seed weight (TSW), flowering time (FlT), and plant height (PH) were measured as described in our previous studies [40, 45, 68, 69]. The main inflorescence length (MIL), primary branch number (PBN), primary branch height (PBH), pod density (PD), bolting time (BoT), budding time (BuT), flowering end time (FET), maturity time (MaT), seed oil content (OIL), and seed protein content (PRO) were measured as described in other studies [70–72].

### Genotypic analyses and genetic linkage map construction

The BnaZN-RIL population of 171 lines was genotyped using the Illumina Infinium 60 K SNP chip, which contains 52,157 SNP markers [73]. Of the 17,286 polymorphic markers, 5405 showed a heterozygous or absent genotype in the parents and were removed from further analysis. Finally, a total of 11,881 high-quality and polymorphic SNP markers were obtained, which were then merged into 4993 co-segregated bins. Genetic linkage mapping was carried out using JoinMap 4.0 software [74] for each of the 19 linkage groups, with the following parameters: goodness of fit was set to ≤ 5.0 with LOD scores > 1.0 and a recombination frequency < 0.4.

### QTL detection and integration

The conditional phenotype value y(T1|T2) was obtained by the mixed model approach for conditional analysis of quantitative traits using QGAStation1.0 (http://ibi.zju.edu.cn/software/qga/index.htm), where T1|T2 indicated that trait 1 was conditioned by trait 2. Conditional and unconditional QTL mapping was performed using the composite interval mapping procedure [75] incorporated in Windows QTL Cartographer 2.5 software. The walk speed, number of control markers, window size and regression method were set to 1 cM, 5, 10 cM and forward regression, respectively. The experiment-wise LOD threshold was determined by permutation test [76] with 1000 repetitions.

The identified QTL of the same trait detected in different environments were integrated into consensus QTL according to the previous report [68]. These consensus QTL were further combined into QTL clusters if their confidence intervals overlapped.

### Construction of trait–QTL network

With slight modification from the previous report [77], traits and QTL were treated as nodes, which were connected by edges using Cytoscape version 3.7.2 software [78]. The QTL were renamed as the chromosome name followed by their positions. If QTL of different traits were integrated into a cluster, it would represent QTL that affect multiple traits. Yield, yield components and yield-related traits are plotted in orange, blue, and green, respectively.

### qRT-PCR and RNA-seq

Total RNA was isolated using the RNeasy Plant Mini Kit (Qiagen). The cDNA was synthesized using the First Strand cDNA Synthesis Kit (Takara). Using the gene-specific primers (Table S11), quantitative reverse-transcription PCR (qRT-PCR) was performed using SYBR^®^ Select Master Mix (2X) according to the manufacturers’ recommendations. The β-*Actin* gene was used as an internal control to normalize transcript levels in both *B. napus* and *A. thaliana* [34]. The relative expression level was calculated using the 2^–ΔΔCt^ method.

RNA-seq was also performed for comparison of gene expression at the transcriptome level. The fragments per kilobase per million reads (FPKM) was used to calculate gene expression levels. DEseq2 (http://bioconductor.org/packages/stats/bioc/DESeq2/) was used to perform gene differential expression analysis. The absolute values of log2 (ratio) ≥ 1 and FDR < 0.05 were chosen as thresholds to screen for DEGs.

### Cloning of full-length sequence of target genes

To verify the sequence variation of target genes in the two parents, their full-length sequences (including the coding region, 5ʹ upstream, and 3ʹ downstream) were amplified from the genomic DNA of Zhongshuang11 and No.73290 by the KOD FX enzyme (cat: KFX-101). The PCR products with expected size were cloned into the *pEASY*^®^-T1 cloning vector (cat: CT101-01)and then sequenced by Beijing Tsingke Biotechnology Co., Ltd.

### Statistical analysis

Variance, correlation and principal component analyses were performed using PROC GLM, CORR, and PRINCOMP procedures incorporated into SAS 9.2 software. Based on the estimated variance components, broad-sense heritability was calculated according to the method described previously [1].

## Fundings

This work was supported by the Agricultural Science and Technology Innovation Program of China (CAAS-ZDRW202105), Natural Science Foundation of China (31,771,840), Agriculture Research System of MOF and MARA of China (CARS-13), Agricultural Science and Technology Innovation Project of China (CAAS-ASTIP-2013-OCRI), and Fundamental Research Funds for Central Non-Profit Institute of Crop Sciences, CAAS (Y2020YJ09).

## Supplementary Information

**Additional file 1**: **Figure S1.** Frequency of distribution for each of the 21 traits investigated in the BnaZN-RIL population planted in six environments. The horizontal and vertical axes are divided with the same spacing, which shows the phenotypic value and line number, respectively. The columns of the different heights represent the number of lines in different groups. Different environments are distinguished by the different colors.

**Additional file 2**: **Figure S2.** Qualitative and quantitative presentation of phenotypic correlation among 21 traits investigated in the BnaZN-RIL population planted in six environments. The abbreviations of 21 traits are shown near both the horizontal and vertical axes. Below the diagonal, the significant correlations among these traits are indicated by circles of different sizes; above the diagonal, the coefficients of significant correlations are shown. The direction and degree of correlation are distinguished by the different colors demonstrated in the legend.

**Additional file 3**: **Figure S3.** Development of high-generation near-isogenic lines for the fine-mapping and evaluation of phenotypic effects. Hybrid F1 was alternatively backcrossed with Zhongshuang11 in Xining and Wuhan. In each generation, foreground selection was performed to choose heterozygous plants (at the target QTL region), which were used for consecutive backcrosses. Finally, the heterozygous BC_12_F_1_ NILs with the highest background recovery rate were self-obsessed to obtain BC_12_F_2_ seeds, for the individual hub-QTL. The homologous BC_12_F_2_ NILs were subjected to phenotypic investigation and compared with the recurrent parent Zhongshuang11.

**Additional file 4**: **Figure S4.** Comparison of the promoter sequence of *BnaA2.FLC* between Zhongshuang11 and No.73290. (A) Alignment of the 2.9 kb sequence in the upstream regulatory region of *BnaA2.FLC* between Zhongshuang11 and No.73290. (B) Structural comparison of the 2.9 sequence in the upstream regulatory region of *BnaA2.FLC* between Zhongshuang11 and No.73290. There were 12 SNPs and two InDels in the promoter region of *BnaA2.FLC* between Zhongshuang11 and No. 73290. Of these, the largest difference was a 10-bp InDel within the core 40-bp motif.

**Additional file 5**: **Figure S5.** Comparison of the *BnaA6.EMB93* sequence between Zhongshuang11 and No.73290. (A) Alignment of the coding sequence of *BnaA6.EMB93* between Zhongshuang11 and No.73290. Dark blue and no fill background represent the consensus and different sequences, respectively. There are a total of 17 SNPs between the two parents. (B) Alignment of the protein sequence of *BnaA6.EMB93* between Zhongshuang11 and No.73290. Only amino acid 228 showed difference between the two parents, which is not within the functional domain of this protein. (C) Structural analysis of the transposon inserted into the promoter region of *BnaA6.EMB93* in No.73290.

**Additional file 6**: **Figure S6.** Quantitative analysis of the expression of *BnaA6.EMB93* and *BnaA9.CYP78A9*. (A) The relative expression level of *BnaA6.EMB93* in the ovaries of Zhongshuang11 and NIL_QC8. The horizontal axis shows buds of different sizes (1-8 mm) before flowering and ovaries at different days after flowering (DAF). (B) The relative expression level of *BnaA9.CYP78A9* in the ovaries of Zhongshuang11 and NIL_QC14. The horizontal axis shows pod walls at two weeks after flowering and seeds at four weeks after flowering. * *P* <0.05, ** *P* <0.01, *** *P* <0.001 (t-test) indicate a significant difference between Zhongshuang11 and NIL. Each data is obtained from three biological replicates.

**Additional file 7**: **Figure S7.** Comparison of the full-length sequence of *BnaA9.CYP78A9* between Zhongshuang11 and NIL_QC14. (A) The full-length genic structure of *BnaA9.CYP78A9 *in Zhongshuang11 and NIL_QC14. There was no difference in the coding sequence, but a 3.6-kb CACTA-like TE insertion into its upstream regulatory region in Zhongshuang11. (B) The alignment of coding sequence of *BnaA9.CYP78A9* in Zhongshuang11 and NIL_QC14.

**Additional file 8**: **Table S1.** Phenotypic variation of both parents and the BnaZN-RIL population in six environments.

**Additional file 9**: **Table S2.** Analysis of variance and estimation of heritability for the 21 traits investigated in the BnaZN-RIL population.

**Additional file 10**: **Table S3.** Summary statistics of the correlation among 21 traits investigated in six environments.

**Additional file 11**: **Table S4.** Three principal components (PC1, PC2, and PC3) in the two locations and six environments.

**Additional file 12**: **Table S5.** Single marker analyses of the highly heterozygous markers on 21 investigated traits.

**Additional file 13**: **Table S6.** Polymorphism of SNPs within the pericentromere region of the 19 chromosomes.

**Additional file 14**: **Table S7.** List of identified QTL (A), consensus QTL (B), and QTL clusters (C).

**Additional file 15**: **Table S8.** Statistical analysis of consensus QTL overlapping between pair-wise combinations among the 21 investigated traits.

**Additional file 16**: **Table S9.** The annotated genes in the fine-mapped genomic regions of four representative hub-QTL clusters.

**Additional file 17**: **Table S10.** The DEGs identified by RNA-seq of SAM in the initial stage of floral bud differentiation between Zhongshuang11 and No.73290.

**Additional file 18**: **Table S11.** List of primers used in this study, including fine-mapping, gene cloning, and qRT-PCR.

## Data Availability

The original datasets of RNA-seq are available in the Sequence Read Archive at the National Center for Biotechnology Information (NCBI) under accession number SRX1715587. The datasets supporting the conclusions of this article are included in the manuscript and additional files.
