# Supplementary figures and images for: Systematic trait dissection in oilseed rape provides a comprehensive view, further insight, and exact roadmap for yield determination
Source: Biotechnol Biofuels Bioprod. 2022 Apr 19;15:38. doi: 10.1186/s13068-022-02134-w (PMC9019968; doi:10.1186/s13068-022-02134-w)

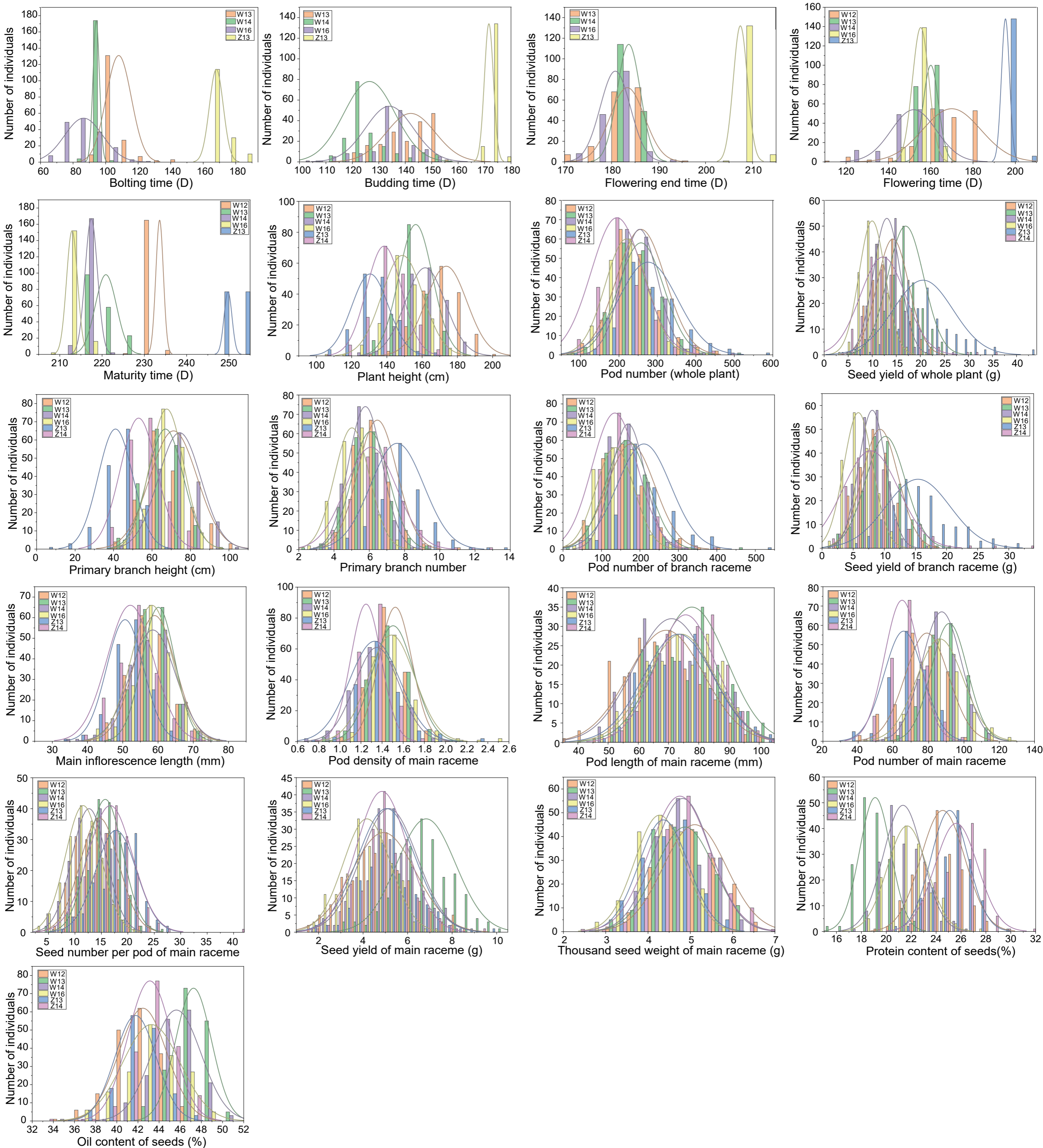

Supplement: Supplementary file 1 — Additional file 1: Figure S1. Frequency of distribution for each of the 21 traits investigated in the BnaZN-RIL population planted in six environments. The horizontal and vertical axes are divided with the same spacing, which shows the phenotypic value and line number, respectively. The columns of the different heights represent the number of lines in different groups. Different environments are distinguished by the different colors. [file 13068_2022_2134_MOESM1_ESM.pdf]

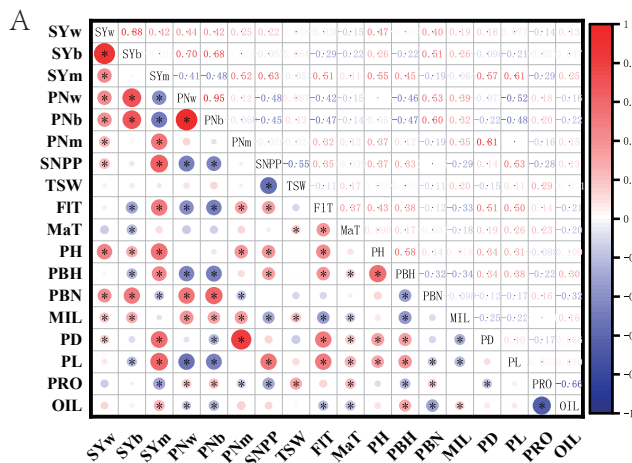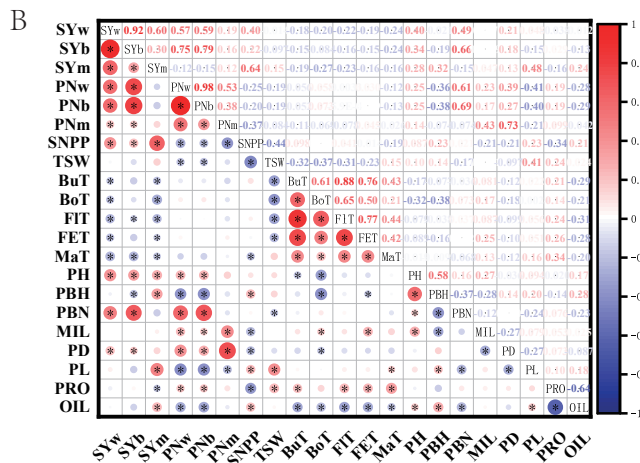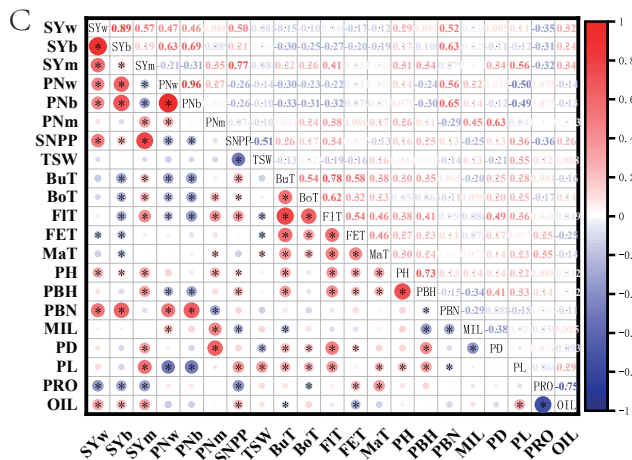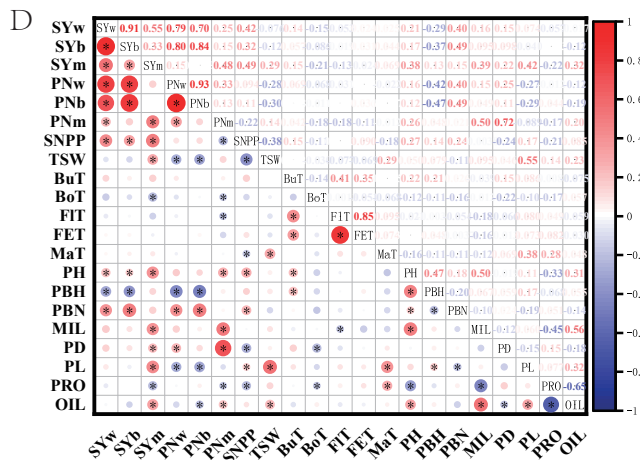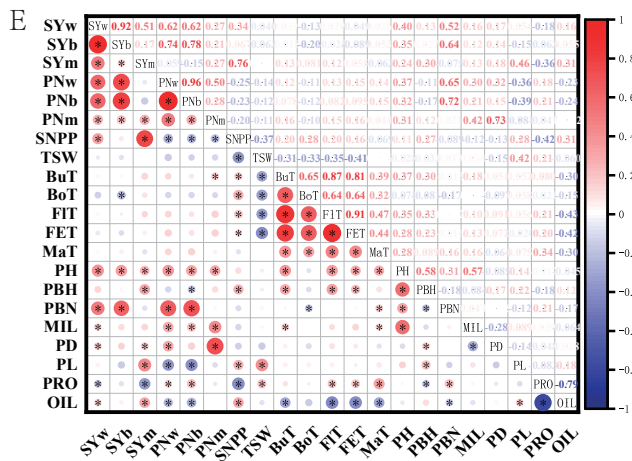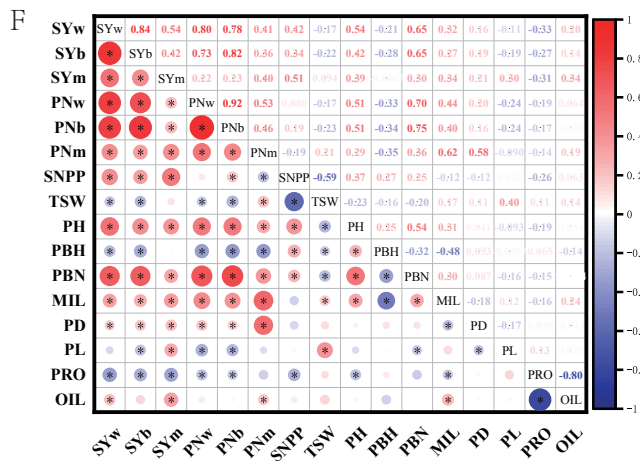

Supplement: Supplementary file 2 — Additional file 2: Figure S2. Qualitative and quantitative presentation of phenotypic correlation among 21 traits investigated in the BnaZN-RIL population planted in six environments. The abbreviations of 21 traits are shown near both the horizontal and vertical axes. Below the diagonal, the significant correlations among these traits are indicated by circles of different sizes; above the diagonal, the coefficients of significant correlations are shown. The direction and degree of correlation are distinguished by the different colors demonstrated in the legend. [file 13068_2022_2134_MOESM2_ESM.pdf]

A

The relative expression level

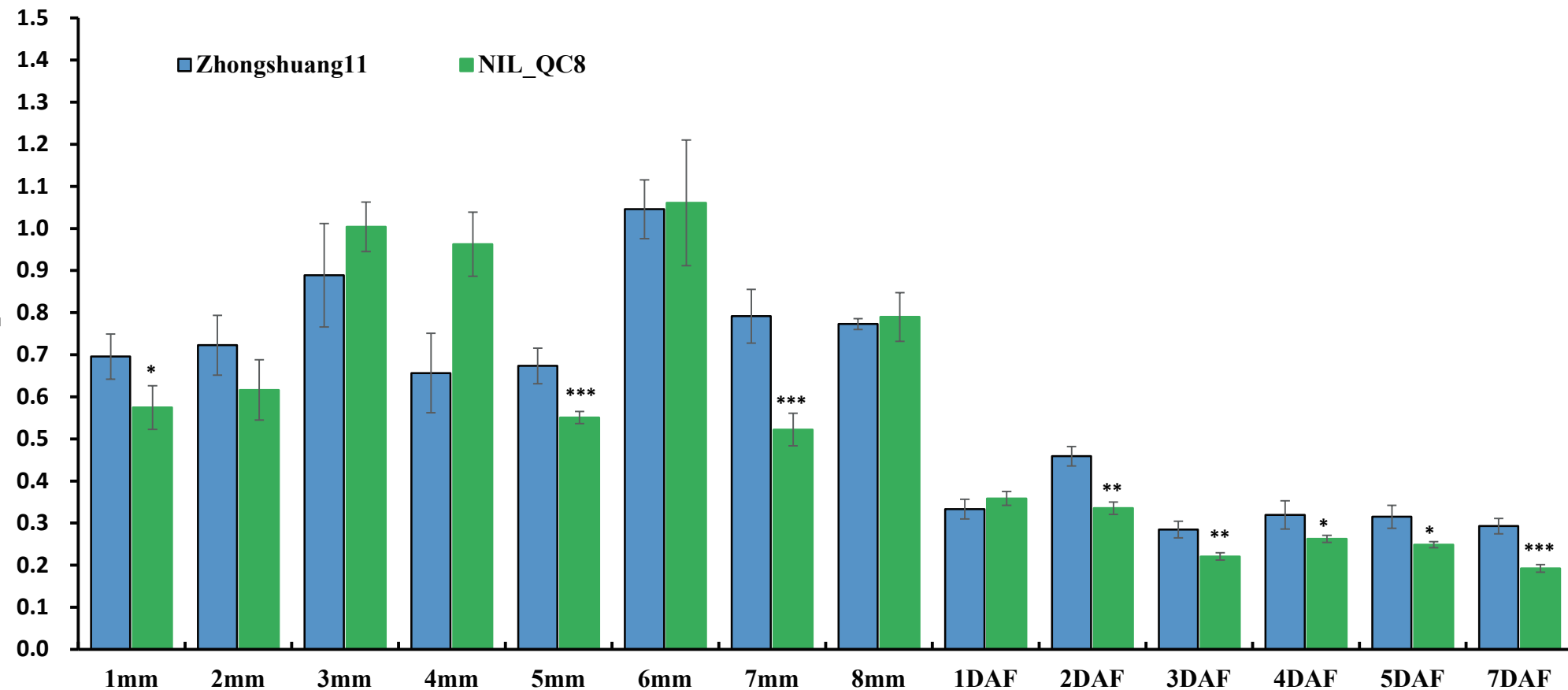

B

The relative expression level

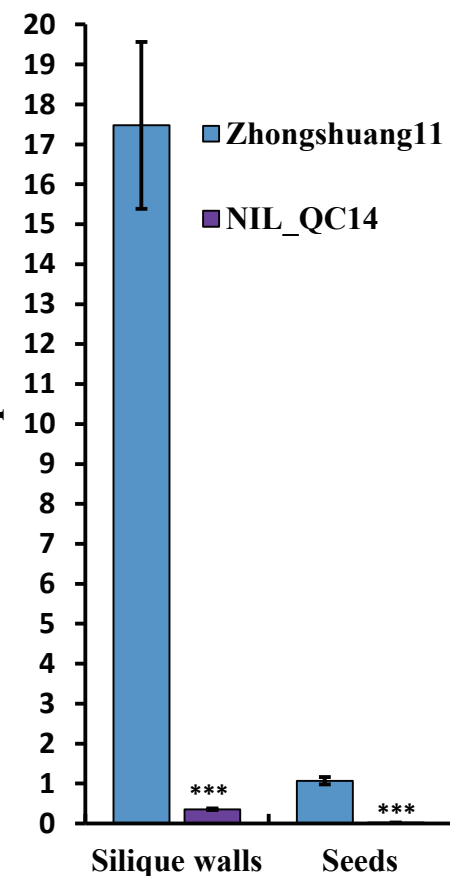

Supplement: Supplementary file 6 — Additional file 6: Figure S6. Quantitative analysis of the expression of BnaA6.EMB93 and BnaA9.CYP78A9. (A) The relative expression level of BnaA6.EMB93 in the ovaries of Zhongshuang11 and NIL_QC8. The horizontal axis shows buds of different sizes (1-8 mm) before flowering and ovaries at different days after flowering (DAF). (B) The relative expression level of BnaA9.CYP78A9 in the ovaries of Zhongshuang11 and NIL_QC14. The horizontal axis shows pod walls at two weeks after flowering and seeds at four weeks after flowering. * P <0.05, ** P <0.01, *** P <0.001 (t-test) indicate a significant difference between Zhongshuang11 and NIL. Each data is obtained from three biological replicates. [file 13068_2022_2134_MOESM6_ESM.pdf]
