# Supplementary material for: Systematic trait dissection in oilseed rape provides a comprehensive view, further insight, and exact roadmap for yield determination
Source: Biotechnol Biofuels Bioprod. 2022 Apr 19;15:38. doi: 10.1186/s13068-022-02134-w (PMC9019968; doi:10.1186/s13068-022-02134-w)
Supplement: Supplementary file 3 — Additional file 3: Figure S3. Development of high-generation near-isogenic lines for the fine-mapping and evaluation of phenotypic effects. Hybrid F1 was alternatively backcrossed with Zhongshuang11 in Xining and Wuhan. In each generation, foreground selection was performed to choose heterozygous plants (at the target QTL region), which were used for consecutive backcrosses. Finally, the heterozygous BC12F1 NILs with the highest background recovery rate were self-obsessed to obtain BC12F2 seeds, for the individual hub-QTL. The homologous BC12F2 NILs were subjected to phenotypic investigation and compared with the recurrent parent Zhongshuang11. [file 13068_2022_2134_MOESM3_ESM.pdf]

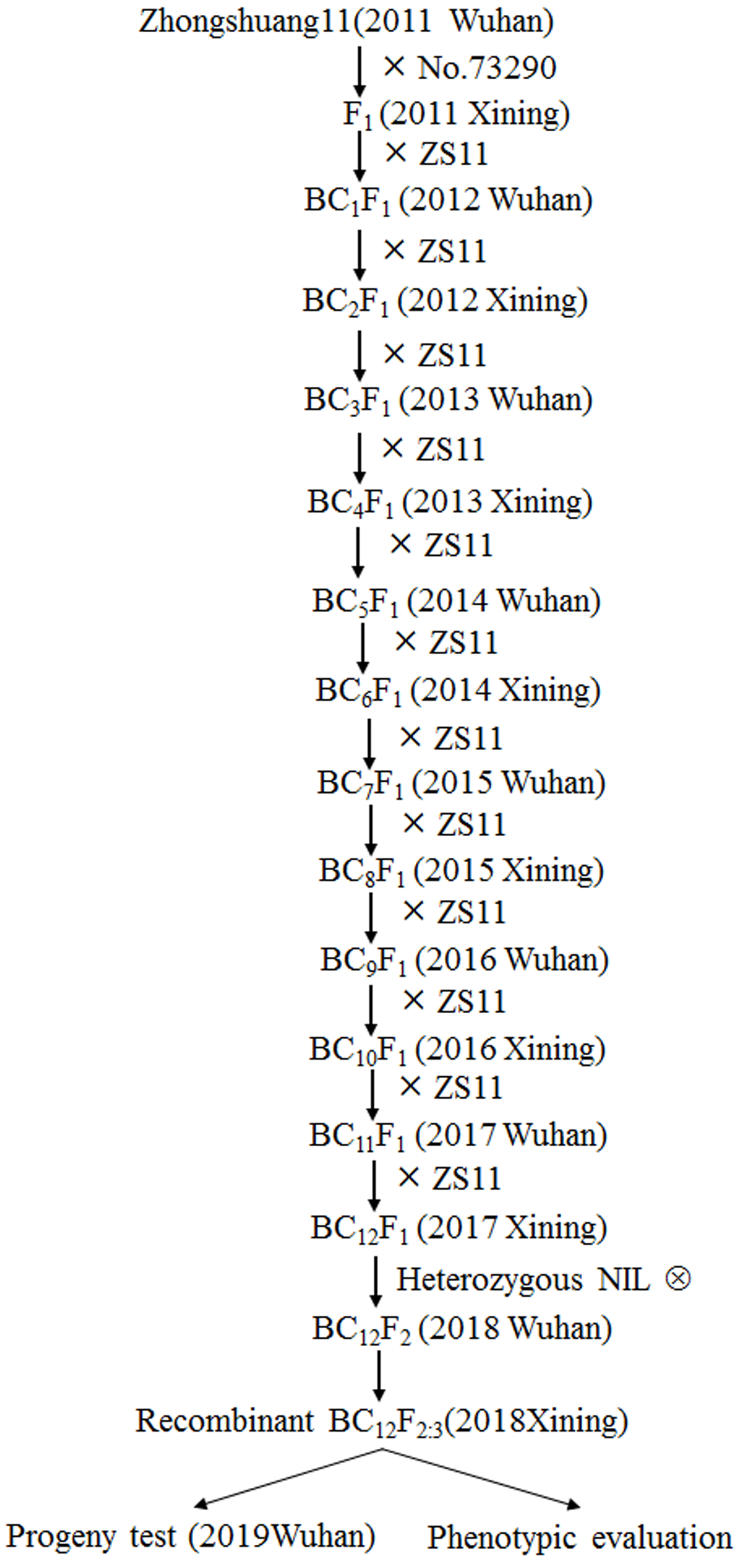
Zhongshuang11(2011 Wuhan)
× No.73290
F1 (2011 Xining)
× ZS11
BC1F1 (2012 Wuhan)
× ZS11
BC2F1 (2012 Xining)
× ZS11
BC3F1 (2013 Wuhan)
× ZS11
BC4F1 (2013 Xining)
× ZS11
BC5F1 (2014 Wuhan)
× ZS11
BC6F1 (2014 Xining)
× ZS11
BC7F1 (2015 Wuhan)
× ZS11
BC8F1 (2015 Xining)
× ZS11
BC9F1 (2016 Wuhan)
× ZS11
BC10F1 (2016 Xining)
× ZS11
BC11F1 (2017 Wuhan)
× ZS11
BC12F1 (2017 Xining)
Heterozygous NIL ⊗
BC12F2 (2018 Wuhan)
Recombinant BC12F2:3(2018Xining)
Progeny test (2019Wuhan)
Phenotypic evaluation
