# Supplementary material for: Systematic trait dissection in oilseed rape provides a comprehensive view, further insight, and exact roadmap for yield determination
Source: Biotechnol Biofuels Bioprod. 2022 Apr 19;15:38. doi: 10.1186/s13068-022-02134-w (PMC9019968; doi:10.1186/s13068-022-02134-w)
Supplement: Supplementary file 4 — Additional file 4: Figure S4. Comparison of the promoter sequence of BnaA2.FLC between Zhongshuang11 and No.73290. (A) Alignment of the 2.9 kb sequence in the upstream regulatory region of BnaA2.FLC between Zhongshuang11 and No.73290. (B) Structural comparison of the 2.9 sequence in the upstream regulatory region of BnaA2.FLC between Zhongshuang11 and No.73290. There were 12 SNPs and two InDels in the promoter region of BnaA2.FLC between Zhongshuang11 and No. 73290. Of these, the largest difference was a 10-bp InDel within the core 40-bp motif. [file 13068_2022_2134_MOESM4_ESM.pdf]

A

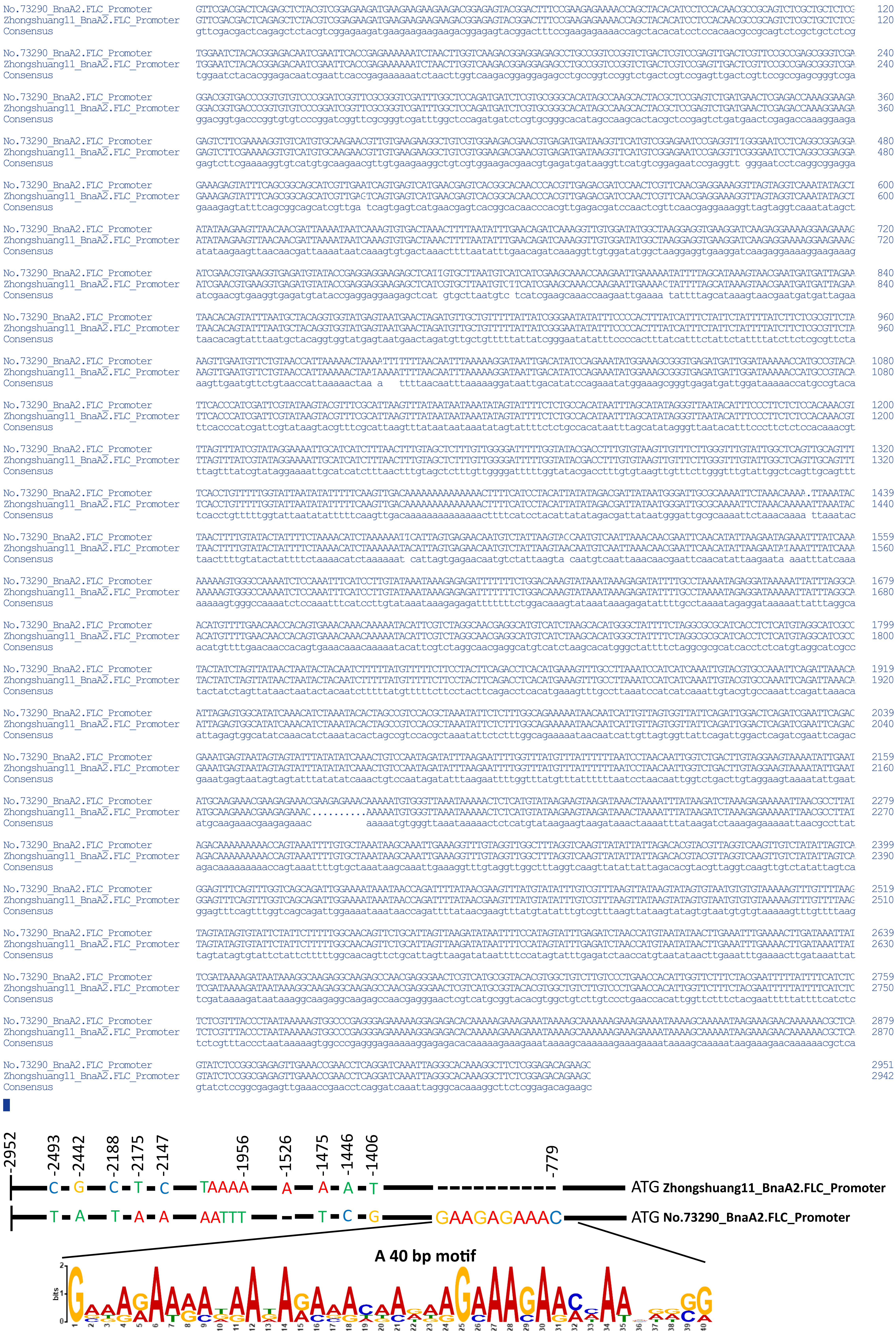
No.73290_BnaA2.FLC_Promoter
Zhongshuang11_BnaA2.FLC_Promoter
Consensus
B
-2952
-2493
-2442
-2188
-2175
-2147
-1956
-1526
-1475
-1446
-1406
-779
ATG Zhongshuang11_BnaA2.FLC_Promoter
ATG No.73290_BnaA2.FLC_Promoter
GAAGAGAAAC
A 40 bp motif
bits
