# Supplementary material for: Systematic trait dissection in oilseed rape provides a comprehensive view, further insight, and exact roadmap for yield determination
Source: Biotechnol Biofuels Bioprod. 2022 Apr 19;15:38. doi: 10.1186/s13068-022-02134-w (PMC9019968; doi:10.1186/s13068-022-02134-w)
Supplement: Supplementary file 5 — Additional file 5: Figure S5. Comparison of the BnaA6.EMB93 sequence between Zhongshuang11 and No.73290. (A) Alignment of the coding sequence of BnaA6.EMB93 between Zhongshuang11 and No.73290. Dark blue and no fill background represent the consensus and different sequences, respectively. There are a total of 17 SNPs between the two parents. (B) Alignment of the protein sequence of BnaA6.EMB93 between Zhongshuang11 and No.73290. Only amino acid 228 showed difference between the two parents, which is not within the functional domain of this protein. (C) Structural analysis of the transposon inserted into the promoter region of BnaA6.EMB93 in No.73290. [file 13068_2022_2134_MOESM5_ESM.pdf]

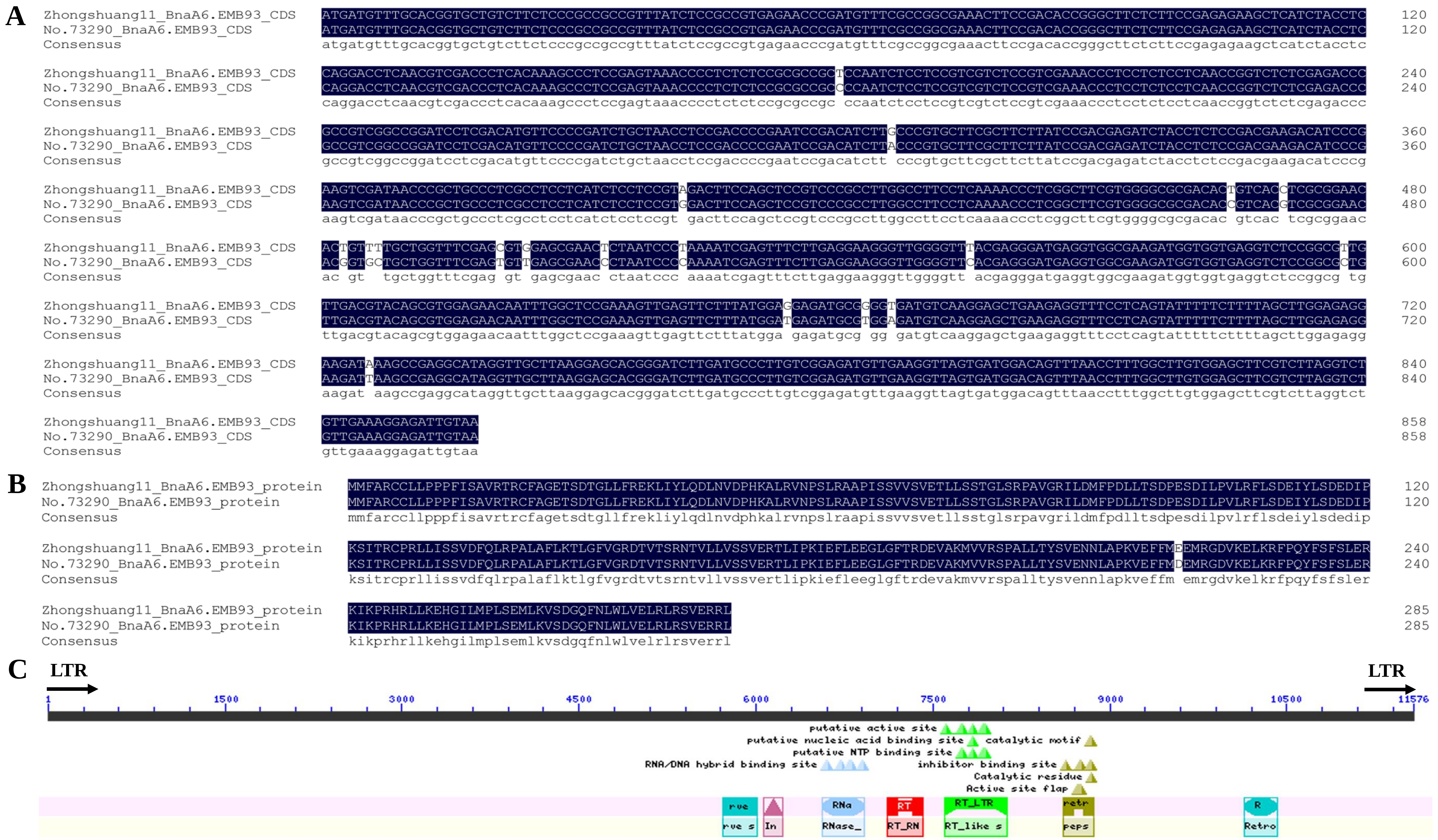
A
Zhongshuang11_BnaA6.EMB93_CDS
No.73290_BnaA6.EMB93_CDS
Consensus
120
240
360
480
600
720
840
858
B
Zhongshuang11_BnaA6.EMB93_protein
No.73290_BnaA6.EMB93_protein
Consensus
120
240
285
C
LTR
LTR
1
1500
3000
4500
6000
7500
9000
10500
11576
putative active site
putative nucleic acid binding site
catalytic motif
putative NTP binding site
RNA/DNA hybrid binding site
inhibitor binding site
Catalytic residue
Active site flap
RNa
RT
RT_LTR
retr
R
In
RNase_
RT_RN
RT_like
peps
Retro
