# Supplementary material for: Systematic trait dissection in oilseed rape provides a comprehensive view, further insight, and exact roadmap for yield determination
Source: Biotechnol Biofuels Bioprod. 2022 Apr 19;15:38. doi: 10.1186/s13068-022-02134-w (PMC9019968; doi:10.1186/s13068-022-02134-w)
Supplement: Supplementary file 7 — Additional file 7: Figure S7. Comparison of the full-length sequence of BnaA9.CYP78A9 between Zhongshuang11 and NIL_QC14. (A) The full-length genic structure of BnaA9.CYP78A9 in Zhongshuang11 and NIL_QC14. There was no difference in the coding sequence, but a 3.6-kb CACTA-like TE insertion into its upstream regulatory region in Zhongshuang11. (B) The alignment of coding sequence of BnaA9.CYP78A9 in Zhongshuang11 and NIL_QC14. [file 13068_2022_2134_MOESM7_ESM.pdf]

A

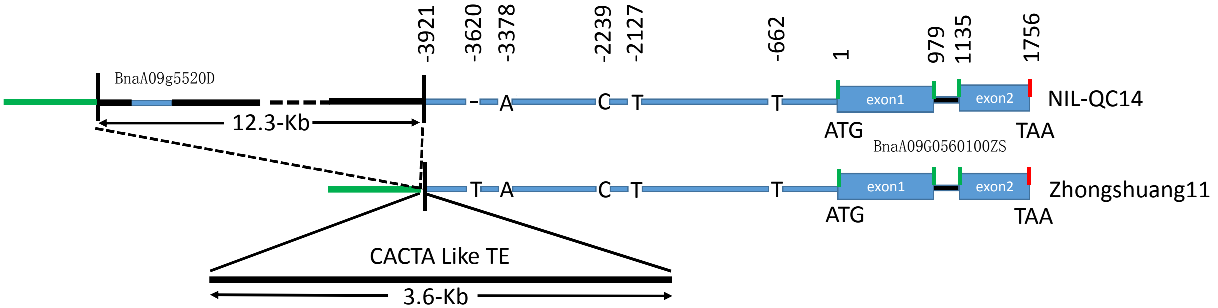

B

```
Zhongshuang11_BnaA09.CYP78A9_CDS  ATGGCGACCAAGCTCGACACAAGCAGCTTACTCTTCGCTCTCTTGTCCAAATGTAGCCTCCTTACTCAAACCCATCTCGCTCTCTCTCTTCTCGTAGCCTCCATGGCTTGCCTCGCTGTC  120
NIL_QC14_BnaA09.CYP78A9_CDS       ATGGCGACCAAGCTCGACACAAGCAGCTTACTCTTCGCTCTCTTGTCCAAATGTAGCCTCCTTACTCAAACCCATCTCGCTCTCTCTCTTCTCGTAGCCTCCATGGCTTGCCTCGCTGTC  120
Consensus                         atggcgaccaagctcgacacaagcagcttactcttcgctctcttgtccaaatgtagcctccttactcaaacccatctcgctctctctcttctcgtagcctccatggcttgcctcgctgtc

Zhongshuang11_BnaA09.CYP78A9_CDS  TCTCTCTTCTACTGGTCTCATCCCGGAGGACCCGCATGGGGAAAATACTTCCTCCATCGCCGTCACCAAACCGCCGTGATTCCCGGACCAAGAGGCTTACCTTTTGTCGGAAGCATGTCT  240
NIL_QC14_BnaA09.CYP78A9_CDS       TCTCTCTTCTACTGGTCTCATCCCGGAGGACCCGCATGGGGAAAATACTTCCTCCATCGCCGTCACCAAACCGCCGTGATTCCCGGACCAAGAGGCTTACCTTTTGTCGGAAGCATGTCT  240
Consensus                         tctctcttctactggtctcatcccggaggacccgcatggggaaaatacttcctccatcgccgtcaccaaaccgccgtgattcccggaccaagaggcttaccttttgtcggaagcatgtct

Zhongshuang11_BnaA09.CYP78A9_CDS  CTCATGTCCAACGCTTTAGCTCACCGCTGCATAGCCGCAGCCGCGGAGAAATTCGGAGCCAAACGTTTAATGGCGTTTAGCTTGGGAGATACTCGCGTGATCGTCACGTGCAACCCTGAT  360
NIL_QC14_BnaA09.CYP78A9_CDS       CTCATGTCCAACGCTTTAGCTCACCGCTGCATAGCCGCAGCCGCGGAGAAATTCGGAGCCAAACGTTTAATGGCGTTTAGCTTGGGAGATACTCGCGTGATCGTCACGTGCAACCCTGAT  360
Consensus                         ctcatgtccaacgctttagctcaccgctgcatagccgcagccgcggagaaattcggagccaaacgtttaatggcgtttagcttgggagatactcgcgtgatcgtcacgtgcaaccctgat

Zhongshuang11_BnaA09.CYP78A9_CDS  GTAGCTAAAGAGATTCTAAACAGTCCGGTTTTCGCTGACCGTCCGGTTAAGGAATCAGCGTATTCCCTTATGTTTAACCGGGCTATCGGTTTCGCTCCTTACGGCGTTTACTGGCGAACG  480
NIL_QC14_BnaA09.CYP78A9_CDS       GTAGCTAAAGAGATTCTAAACAGTCCGGTTTTCGCTGACCGTCCGGTTAAGGAATCAGCGTATTCCCTTATGTTTAACCGGGCTATCGGTTTCGCTCCTTACGGCGTTTACTGGCGAACG  480
Consensus                         gtagctaaagagattctaaacagtccggttttcgctgaccgtccggttaaggaatcagcgtattcccttatgtttaaccgggctatcggtttcgctccttacggcgtttactggcgaacg

Zhongshuang11_BnaA09.CYP78A9_CDS  TTGAGGAAAATCGCGTCTAATCATCTTTTCAGCCCGAAACAGATCAAACGCTCTGAAACGCAGAGACGTGTGATCGCGAATCAGATCGTGAAATGTCTCGAGAAACAGAGCAGCGGCAAC  600
NIL_QC14_BnaA09.CYP78A9_CDS       TTGAGGAAAATCGCGTCTAATCATCTTTTCAGCCCGAAACAGATCAAACGCTCTGAAACGCAGAGACGTGTGATCGCGAATCAGATCGTGAAATGTCTCGAGAAACAGAGCAGCGGCAAC  600
Consensus                         ttgaggaaaatcgcgtctaatcatcttttcagcccgaaacagatcaaacgctctgaaacgcagagacgtgtgatcgcgaatcagatcgtgaaatgtctcgagaaacagagcagcggcaac

Zhongshuang11_BnaA09.CYP78A9_CDS  GAAGGACTCTGTTTTGCTCGTGACTTGATCAAAACGGCATCGCTTAATAGCATGATGTGCTCTGTTTTCGGGAAAGAGTACGAGCTTGAGCATGAACACGATGAAGTTAATGAGCTTCGT  720
NIL_QC14_BnaA09.CYP78A9_CDS       GAAGGACTCTGTTTTGCTCGTGACTTGATCAAAACGGCATCGCTTAATAGCATGATGTGCTCTGTTTTCGGGAAAGAGTACGAGCTTGAGCATGAACACGATGAAGTTAATGAGCTTCGT  720
Consensus                         gaaggactctgttttgctcgtgacttgatcaaaacggcatcgcttaatagcatgatgtgctctgttttcgggaaagagtacgagcttgagcatgaacacgatgaagttaatgagcttcgt

Zhongshuang11_BnaA09.CYP78A9_CDS  GGTTTGGTCGAAGAAGGTTATGATTTACTCGGAACACTTAACTGGACCGATCATCTCCCGTGGCTGTCGGAATTTGATCCTCAGAGAATCCGGTCTAGATGTTCTAATCTTGTACCTAAA  840
NIL_QC14_BnaA09.CYP78A9_CDS       GGTTTGGTCGAAGAAGGTTATGATTTACTCGGAACACTTAACTGGACCGATCATCTCCCGTGGCTGTCGGAATTTGATCCTCAGAGAATCCGGTCTAGATGTTCTAATCTTGTACCTAAA  840
Consensus                         ggtttggtcgaagaaggttatgatttactcggaacacttaactggaccgatcatctcccgtggctgtcggaatttgatcctcagagaatccggtctagatgttctaatcttgtacctaaa

Zhongshuang11_BnaA09.CYP78A9_CDS  GTAAACCGGTTCGTGAACCGGATTATCTCTGACCACCGTGATCAAACTCGTGACTCGCCTAGCGACTTCGTTGACGTATTGCTCTCTCTCGATGGTCAGGATAAGTTATCTGACCCGGAT  960
NIL_QC14_BnaA09.CYP78A9_CDS       GTAAACCGGTTCGTGAACCGGATTATCTCTGACCACCGTGATCAAACTCGTGACTCGCCTAGCGACTTCGTTGACGTATTGCTCTCTCTCGATGGTCAGGATAAGTTATCTGACCCGGAT  960
Consensus                         gtaaaccggttcgtgaaccggattatctctgaccaccgtgatcaaactcgtgactcgcctagcgacttcgttgacgtattgctctctctcgatggtcaggataagttatctgacccggat

Zhongshuang11_BnaA09.CYP78A9_CDS  ATGGTCGCCGTTCTCTGGGAAATGATATTCAGAGGAACTGACACGGTGGCTGTCTTGATCGAGTGGGTTCTTGCTAGGATGGTCCTTCATCCAGATATTCAGTCAATGGTTCACAACGAG  1080
NIL_QC14_BnaA09.CYP78A9_CDS       ATGGTCGCCGTTCTCTGGGAAATGATATTCAGAGGAACTGACACGGTGGCTGTCTTGATCGAGTGGGTTCTTGCTAGGATGGTCCTTCATCCAGATATTCAGTCAATGGTTCACAACGAG  1080
Consensus                         atggtcgccgttctctgggaaatgatattcagaggaactgacacggtggctgtcttgatcgagtgggttcttgctaggatggtccttcatccagatattcagtcaatggttcacaacgag

Zhongshuang11_BnaA09.CYP78A9_CDS  CTTGATCAGAACGTGGGAAGATCAAGAACCGTGGAAGAATCTGACGTGGCGTCTCTTACATATCTGACTGCTGTTGTGAAAGAAGTCTTGAGGCTTCACCCGCCAGGCCCACTTCTGTCG  1200
NIL_QC14_BnaA09.CYP78A9_CDS       CTTGATCAGAACGTGGGAAGATCAAGAACCGTGGAAGAATCTGACGTGGCGTCTCTTACATATCTGACTGCTGTTGTGAAAGAAGTCTTGAGGCTTCACCCGCCAGGCCCACTTCTGTCG  1200
Consensus                         cttgatcagaacgtgggaagatcaagaaccgtggaagaatctgacgtggcgtctcttacatatctgactgctgttgtgaaagaagtcttgaggcttcacccgccaggcccacttctgtcg

Zhongshuang11_BnaA09.CYP78A9_CDS  TGGGCCCGCTTAGCAATCACAGACACAATCATTGACGGCCGTCGTGTACCGGCAGGGACCACCGCAATGGTGAACATGTGGGCTATAGCACATGACCCACAAGTGTGGGAAAATCCTTTG  1320
NIL_QC14_BnaA09.CYP78A9_CDS       TGGGCCCGCTTAGCAATCACAGACACAATCATTGACGGCCGTCGTGTACCGGCAGGGACCACCGCAATGGTGAACATGTGGGCTATAGCACATGACCCACAAGTGTGGGAAAATCCTTTG  1320
Consensus                         tgggcccgcttagcaatcacagacacaatcattgacggccgtcgtgtaccggcagggaccaccgcaatggtgaacatgtgggctatagcacatgacccacaagtgtgggaaaatcctttg

Zhongshuang11_BnaA09.CYP78A9_CDS  GAGTTTAAACCTGAACGGTTTGTAGCAAAGGAAGGTGAGGTCGAGTTCTCGGTTCTTGGTTCGGATCTGAGGCTAGCACCGTTCGGGTCGGGCCGTCGGGTCTGCCCTGGAAAGAATCTT  1440
NIL_QC14_BnaA09.CYP78A9_CDS       GAGTTTAAACCTGAACGGTTTGTAGCAAAGGAAGGTGAGGTCGAGTTCTCGGTTCTTGGTTCGGATCTGAGGCTAGCACCGTTCGGGTCGGGCCGTCGGGTCTGCCCTGGAAAGAATCTT  1440
Consensus                         gagtttaaacctgaacggtttgtagcaaaggaaggtgaggtcgagttctcggttcttggttcggatctgaggctagcaccgttcgggtcgggccgtcgggtctgccctggaaagaatctt

Zhongshuang11_BnaA09.CYP78A9_CDS  GGTTTGTCCACCGTGATGTATTGGATCGCTACTCTTATGCACGAGTTTGAGTGGTTTGCACCTACAGGTGAAAAGACTGTTGACTTGTCCGAGAAACTGAGGCTCTCGTGTGAGATGGCT  1560
NIL_QC14_BnaA09.CYP78A9_CDS       GGTTTGTCCACCGTGATGTATTGGATCGCTACTCTTATGCACGAGTTTGAGTGGTTTGCACCTACAGGTGAAAAGACTGTTGACTTGTCCGAGAAACTGAGGCTCTCGTGTGAGATGGCT  1560
Consensus                         ggtttgtccaccgtgatgtattggatcgctactcttatgcacgagtttgagtggtttgcacctacaggtgaaaagactgttgacttgtccgagaaactgaggctctcgtgtgagatggct

Zhongshuang11_BnaA09.CYP78A9_CDS  AATCCTCTTGCTGTTAAACTGAGCGGCAGGCGCGGTTAA  1599
NIL_QC14_BnaA09.CYP78A9_CDS       AATCCTCTTGCTGTTAAACTGAGCGGCAGGCGCGGTTAA  1599
Consensus                         aatcctcttgctgttaaactgagcggcaggcgcggttaa
```
